# Compensating for population sampling in simulations of epidemic spread on temporal contact networks

**DOI:** 10.1038/ncomms9860

**Published:** 2015-11-13

**Authors:** Mathieu Génois, Christian L. Vestergaard, Ciro Cattuto, Alain Barrat

**Affiliations:** 1 Aix Marseille Université, Université de Toulon, CNRS, CPT, UMR 7332, 13288 Marseille, France; 2 Data Science Laboratory, ISI Foundation, 10126 Torino, Italy

## Abstract

Data describing human interactions often suffer from incomplete sampling of the underlying population. As a consequence, the study of contagion processes using data-driven models can lead to a severe underestimation of the epidemic risk. Here we present a systematic method to alleviate this issue and obtain a better estimation of the risk in the context of epidemic models informed by high-resolution time-resolved contact data. We consider several such data sets collected in various contexts and perform controlled resampling experiments. We show how the statistical information contained in the resampled data can be used to build a series of surrogate versions of the unknown contacts. We simulate epidemic processes on the resulting reconstructed data sets and show that it is possible to obtain good estimates of the outcome of simulations performed using the complete data set. We discuss limitations and potential improvements of our method.

## Abstract (web summary)

Data describing human interaction suffer from incomplete sampling of population, affecting the reliability of studies on contagion spreading. Here, the authors present a systematic method to resample data and generate surrogate datasets of the unknown contacts, which approximate well the complete datasets.

## Graphical abstract

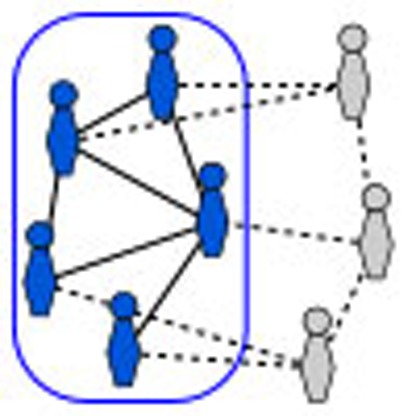

Human interactions play an important role in determining the potential transmission routes of infectious diseases and other contagion phenomena^1^. Their measure and characterization represent an invaluable contribution to the study of transmissible diseases^2^. While surveys and diaries in which volunteer participants record their encounters^3^,^4^,^5^,^6^,^7^ have provided crucial insights (see, however, (refs 4, 8, 9) for recent investigations of the memory biases inherent in self-reporting procedures), new approaches have recently emerged to measure contact patterns between individuals with high resolution, using wearable sensors that can detect the proximity of other similar devices^10^,^11^,^12^,^13^,^14^,^15^,^16^,^17^,^18^,^19^,^20^. The resulting measuring infrastructures register contacts specifically within the closed population formed by the participants wearing sensors, with high spatial and temporal resolutions. In the recent years, several data gathering efforts have used such methods to obtain, analyse and publish data sets describing the contact patterns between individuals in various contexts in the form of temporal networks^14^,^20^,^21^,^22^,^23^,^24^: nodes represent individuals and, at each time step, a link is drawn between pairs of individuals who are in contact^25^. Such data has been used to inform models of epidemic spreading phenomena used to evaluate epidemic risks and mitigation strategies in specific, size-limited contexts such as schools or hospitals^14^,^19^,^20^,^22^,^26^,^27^,^28^,^29^,^30^,^31^,^32^, finding in particular outcomes consistent with observed outbreak data^20^ or providing evidence of links between specific contacts and transmission events^19^,^32^.

Despite the relevance and interest of such detailed data sets, they suffer from the intrinsic limitation of the data gathering method: contacts are registered only between participants wearing sensors. Contacts with and between individuals who do not wear sensors are thus missed. In other words, as most often not all individuals accept to participate by wearing sensors, many data sets obtained by such techniques suffer from population sampling, despite efforts to maximise participation through for example, scientific engagement of participants^24^,^33^. Hence, the collected data only contains information on contacts occurring among a fraction of the population under study.

Population sampling is well known to affect the properties of static networks^34^,^35^,^36^: various statistical properties and mixing patterns of the contact network of a fraction of the population of interest may differ from those of the whole population, even if the sampling is uniform^37^,^38^,^39^,^40^, and several works have focused on inferring network statistics from the knowledge of incomplete network data^39^,^41^,^42^,^43^,^44^. Both structural and temporal properties of time-varying networks might as well be affected by missing data effects^16^,^39^.

In addition, a crucial though little studied consequence of such missing data is that simulations of dynamical processes in data-driven models can be affected if incomplete data are used^38^,^39^,^45^. For instance, in simulations of epidemic spreading, excluded nodes are by definition unreachable and thus equivalent to immunised nodes. Due to herd vaccination effects, the outcome of simulations of epidemic models on sampled networks will thus be underestimated with respect to simulations on the whole network (Note, however, that in the context of transportation networks, the inclusion of the most important transportation nodes can be sufficient to describe the global worldwide spread of influenza-like illnesses, at least in terms of times of arrival of the spread^45^.) How to estimate the outcome of dynamical processes on contact networks using incomplete data remains an open question.

Here we make progresses on this issue for incompletely sampled data describing networks of human face-to-face interactions, collected by infrastructures based on sensors, under the assumption that the population participating to the data collection is a uniform random sample of the whole population of interest. (We do not therefore address here the issue of non-uniform sampling of contacts that may result from other measurement methods such as diaries or surveys.) We proceed through resampling experiments on empirical data sets in which we exclude uniformly at random a fraction of the individuals (nodes of the contact network). We measure how relevant network statistics vary under such uniform resampling and confirm that, although some crucial properties are stable, numerical simulations of spreading processes performed using incomplete data lead to strong underestimations of the epidemic risk. Our goal and main contribution consists in putting forward and comparing a hierarchy of systematic methods to provide better estimates of the outcome of models of epidemic spread in the whole population under study. To this aim, we do not try to infer the true sequence of missing contacts. Instead, the methods we present consist in the construction of surrogate contact sequences for the excluded nodes, using only structural and temporal information available in the resampled contact data. We perform simulations of spreading processes on the reconstructed data sets, obtained by the union of the resampled and surrogate contacts, and investigate how their outcomes vary depending on the amount of information included in the reconstruction method. We show that it is possible to obtain outcomes close to the results obtained on the complete data set, while, as mentioned above, using only the incomplete data severely underestimates the epidemic risk. We show the efficiency of our procedure using three data sets collected in widely different contexts and representative of very different population structures found in day-to-day life: a scientific conference, a high school and a workplace. We finally discuss the limitations of our method in terms of sampling range, model parameters and population sizes.

## Results

### Data and methodology

We consider data sets describing contacts between individuals, collected by the SocioPatterns collaboration (http://www.sociopatterns.org) in three different settings: a workplace (office building, InVS)^46^, a high school (Thiers13)^24^ and a scientific conference (SFHH)^21^,^22^. These data correspond to the close face-to-face proximity of individuals equipped with wearable sensors, at a temporal resolution of 20 s (ref. 16). Table 1 summarises the characteristics of each data set. The contact data are represented by temporal networks, in which nodes represent the participating individuals and a link between two nodes *i* and *j* at time *t* indicates that the two corresponding persons were in contact at that time. These three data sets were chosen as representative of different types of day-to-day contexts and of different contact network structures: the SFHH data correspond to a rather homogeneous contact network; the InVS and Thiers13 populations were instead structured in departments and classes, respectively. Moreover, high school classes (Thiers13) are of similar sizes while the InVS department sizes are unequal. Finally, the high school contact patterns are constrained by strict and repetitive school schedules, while contacts in offices are less regular across days.

To quantify how the incompleteness of data, assumed to stem from a uniformly random participation of individuals to the data collection, affects the outcome of simulations of dynamical processes, we consider as ground truth the available data and perform population resampling experiments by removing a fraction *f* of the nodes uniformly at random. (Note that the full data sets are also samples of all the contacts that occurred in the populations, as the participation rate was lower than 100% in each case.) We then simulate on the resampled data the paradigmatic susceptible-infectious-recovered (SIR) and the susceptible-infectious-susceptible (SIS) models of epidemic propagation. In these models, a susceptible (S) node becomes infectious (I) at rate *β* when in contact with an infectious node. Infectious nodes recover spontaneously at rate *μ*. In the SIR model, nodes then enter an immune recovered (R) state, while in the SIS model, nodes become susceptible again and can be reinfected. The quantities of interest are for the SIR model the distribution of epidemic sizes, defined as the final fraction of recovered nodes, and for the SIS model the average fraction of infectious nodes *i*_∞_ in the stationary state. We also calculate for the SIR model the fraction of epidemics that infect more than 20% of the population and the average size of these epidemics. For the SIS model, we determine the epidemic threshold **β**_c_ for different values of *μ*: it corresponds to the value of *β* that separates an epidemic-free state (*i*_∞_=0) for **β**<**β**_c_ from an endemic state (*i*_∞_>0) for **β**>**β**_c_, and is thus an important indicator of the epidemic risk. We refer to the Methods section for further details on the simulations.

We then present several methods for constructing surrogate data using only information contained in the resampled data. We compare for each data set the outcomes of simulations performed on the whole data set, on resampled data sets with a varying fraction of nodes removed, *f*, and on the reconstructed data sets built using these various methods.

### Uniformly resampled contact networks

Missing data are known to affect the various properties of contact networks in different ways. In particular, the number of neighbours (degree) of a node decreases as the fraction *f* of removed nodes increases, since removing nodes also removes links to these nodes. Under the hypothesis of uniform sampling, the average degree 〈*k*〉 becomes (1−*f*)〈*k*〉 for the resampled network^47^. As a result, the density of the resampled aggregated contact network, defined as the number of links divided by the total number of possible links between the nodes, does not depend on *f*. The same reasoning applies to the density *ρ*_AB_ of links between groups of nodes A and B, defined as the number of links *E*_AB_ between nodes of group A and nodes of group B, normalised by the maximum possible number of such links, *n*_A_*n*_B_, where *n*_A_ is the number of nodes of group A (for A=B, the maximum possible number of links is *n*_A_(*n*_A_−1)/2): both the expected number of neighbours of group B for nodes of group A (given by *E*_AB_/*n*_A_) and the number *n*_B_ of nodes in group B are indeed reduced by a factor (1−*f*), so that *ρ*_AB_ remains constant. This means that the link density contact matrix, which gathers these densities and gives a measure of the interaction between groups (here classes or departments), is stable under uniform resampling. We illustrate these results on our empirical data sets in Supplementary Figs 1–4 (see also Supplementary Note 1). Table 2 and Supplementary Fig. 2 show in particular that the similarities between the original and resampled matrices are high for all data sets (Supplementary Figs 3–4 for the contact matrices themselves).

Finally, the temporal statistics of the contact network are not affected by population sampling, as noted in ref. 16 for other data sets: the distributions of contact and inter-contact durations (the inter-contact durations are the times between consecutive contacts on a link), of number of contacts per link and of cumulated contact durations (that is, of the link weights in the aggregated network) do not change when the network is sampled uniformly (Supplementary Fig. 1). For structured populations, an interesting property is moreover illustrated in Supplementary Figs 5–6: although the distributions of contact durations occurring between members of the same group or between individuals belonging to different groups are indistinguishable, this is not the case for the distributions of the numbers of contacts per link nor, as a consequence, for the distributions of cumulated contact durations. In fact, both cumulated contact durations and numbers of contacts per link are more broadly distributed for links joining members of the same group. The figures show that this property is stable under uniform resampling.

**Figure 1:**
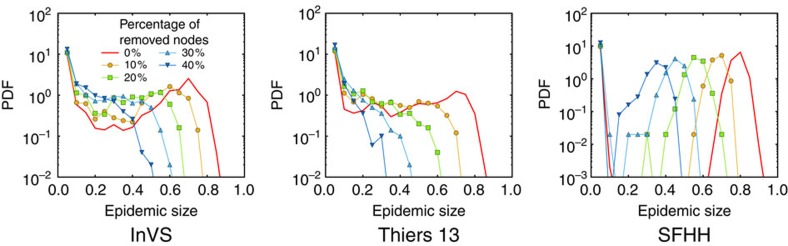
SIR epidemic simulations on resampled contact networks. We plot the distributions of epidemic sizes (fraction of recovered individuals) at the end of SIR processes simulated on top of resampled contact networks, for different values of the fraction *f* of nodes removed. The plot shows the progressive disparition of large epidemic outbreaks as *f* increases. The parameters of the SIR models are *β*=0.0004 and *β*/*μ*=1,000 (InVS) or *β*/*μ*=100 (Thiers13 and SFHH). The case *f*=0 corresponds to simulations using the whole data set, that is, the reference case. For each value of *f*, 1,000 independent simulations were performed.

Despite the robustness of these properties, the outcome of simulations of epidemic spread is strongly affected by the resampling. As Fig. 1 illustrates, the probability of large outbreaks in the SIR model decreases strongly as *f* increases and even vanishes at large *f*. As mentioned above, such a result is expected, since the removed nodes act as if they were immunised: sampling hinders the propagation in simulations by removing transmission routes between the remaining nodes. As a consequence, the prevalence and the final size of the outbreaks are systematically underestimated by simulations of the SIR model on the resampled network with respect to simulations on the whole data set (for the SIS model, the epidemic threshold is overestimated): resampling leads overall to a systematic underestimation of the epidemic risk, and Fig. 1 illustrates the extent of this underestimation.

### Estimation of epidemic sizes

We now present a series of methods to improve the estimation of the epidemic risk in simulations of epidemic spread on temporal network data sets in which nodes (individuals) are missing uniformly at random. Note that we do not address here the problem of link prediction^48^ as our aim is not to infer the missing contacts. The hierarchy of methods we put forward uses increasing amounts of information corresponding to increasing amounts of detail on the group and temporal structure of the contact patterns, as measured in the resampled network. We moreover assume that the timelines of scheduled activity are known (that is, nights and weekends, during which no contact occurs).

For each data set, considered as ground truth, we create resampled data sets by removing at random a fraction *f* of the *N* nodes. We then measure on each resampled data set a series of statistics of the resulting contact network and construct stochastic, surrogate versions of the missing part of the network by creating for each missing node a surrogate instance of its links and a synthetic timeline of contacts on each surrogate link, in the different ways described below (Supplementary Methods and Methods section for more details on their practical implementation).

Method 0. The first effect of missing data is to decrease the average degree of the aggregate contact network, while keeping its density constant. Hence, the simplest approach is to merely compensate this decrease. We therefore measure the density of the resampled contact network *ρ*_s_, as well as the average aggregate duration of the contacts, 〈*w*〉_s_. We then add back the missing nodes and create surrogate links between these nodes and between these nodes and the nodes of the resampled data set at random, with the only constraint to keep the overall link density fixed to *ρ*_s_. We attribute to each surrogate link the same weight 〈*w*〉_s_ and create for each link a timeline of randomly chosen contact events of equal length Δ*t*=20 s (the temporal resolution of the data set) whose total duration gives back 〈*w*〉_s_.

Method W. The heterogeneity of aggregated contact durations is known to play a role in the spreading patterns of model diseases^4^,^20^,^22^,^49^. We therefore refine Method 0 by collecting in the resampled data the list {*w*} of aggregate contact durations, or weights (W). We build the surrogate links and surrogate timelines of contacts on each link as in Method 0, except that each surrogate link carries a weight extracted at random from {*w*}, instead of the average 〈*w*〉_s_.

Method WS. The fact that the population is divided into groups of individuals such as classes or departments can have a strong impact on the structure of the contact network^20^,^23^ and on spreading processes^50^. We thus measure the link density contact matrix of the resampled data, and construct surrogate links in a way to keep this matrix fixed (equal to the value measured in the resampled data), in the spirit of stochastic block models with fixed numbers of edges between blocks^51^. Moreover, we collect in the resampled data two separate lists of aggregate contact durations: {*w*}^int^ gathers the weights of links between individuals belonging to the same group, and {*w*}^ext^ is built with the weights of links joining individuals of different groups. For each surrogate link, its weight is extracted at random either from {*w*}^int^ if it joins individuals of the same group or from {*w*}^ext^ if it associates individuals of different groups. Timelines are then attributed to links as in W. This method assumes that the number of missing nodes in each group is known, and preserves the group structure (S) of the population.

Method WT. Several works have investigated how the temporal characteristics of networks can slow down or accelerate spreading^25^,^30^,^52^. To take these characteristics into account, we measure in the resampled data the distributions of number of contacts per link and of contact and inter-contact durations, in addition to the global network density. We build surrogate links as in Method W, and construct on each link a synthetic timeline in a way to respect the measured temporal statistics (T) of contacts. More precisely, we attribute at random a number of contacts (taken from the measured distribution) to each surrogate link, and then alternate contact and inter-contact durations taken at random from the respective empirical distributions.

Method WST. This method conserves the distribution of link weights (W), the group structure (S), and the temporal characteristics of contacts (T): surrogate links are built and weights assigned as in method WS, and contact timelines on each link as in method WT.

Each of these methods uses a different amount of information gathered from the resampled data. Methods 0, W and WT include an increasing amount of detail on the temporal structure of contacts: method 0 assumes homogeneity of aggregated contact durations, while W takes into account their heterogeneity, and WT reproduces heterogeneities of contact and inter-contact durations. On the other hand, neither of these three methods assume any knowledge of the population group structure. This can be due either to an effective complete lack of knowledge about the population structure, as in the SFHH data, or to the lack of data on the repartition of the missing nodes in the groups. Methods WS and WST on the other hand reproduce the group structure as in a stochastic block model with fixed number of links within and between groups, and take into account the difference between the distributions of numbers of contacts and aggregate durations between individuals of the same or of different groups. Indeed, links within groups correspond on average to larger weights, as found empirically in ref. 50 and discussed above (Supplementary Figs 5–6). Overall, method WST is the one that uses most information measured in the resampled data. Additional properties such as the transitivity–also stable under resampling procedure, see Supplementary Fig. 7–can also be measured in the resampled data and imposed in the construction of surrogate links, as detailed in the Supplementary Methods. This comes, however, at a strong computational cost and we have verified that it does not impact significantly our results, as shown in the Supplementary Fig. 8.

We check in Table 2, Supplementary Note 2 and Supplementary Figs 9–14 that the statistical properties of the resulting reconstructed (surrogate) networks, obtained by the union of the resampled data and of the surrogate links, are similar to the ones of the original data for the WST method. We emphasise again that our aim is not to infer the true missing contacts, so that we do not compare the detailed structures of the surrogate and original contact networks.

**Figure 2:**
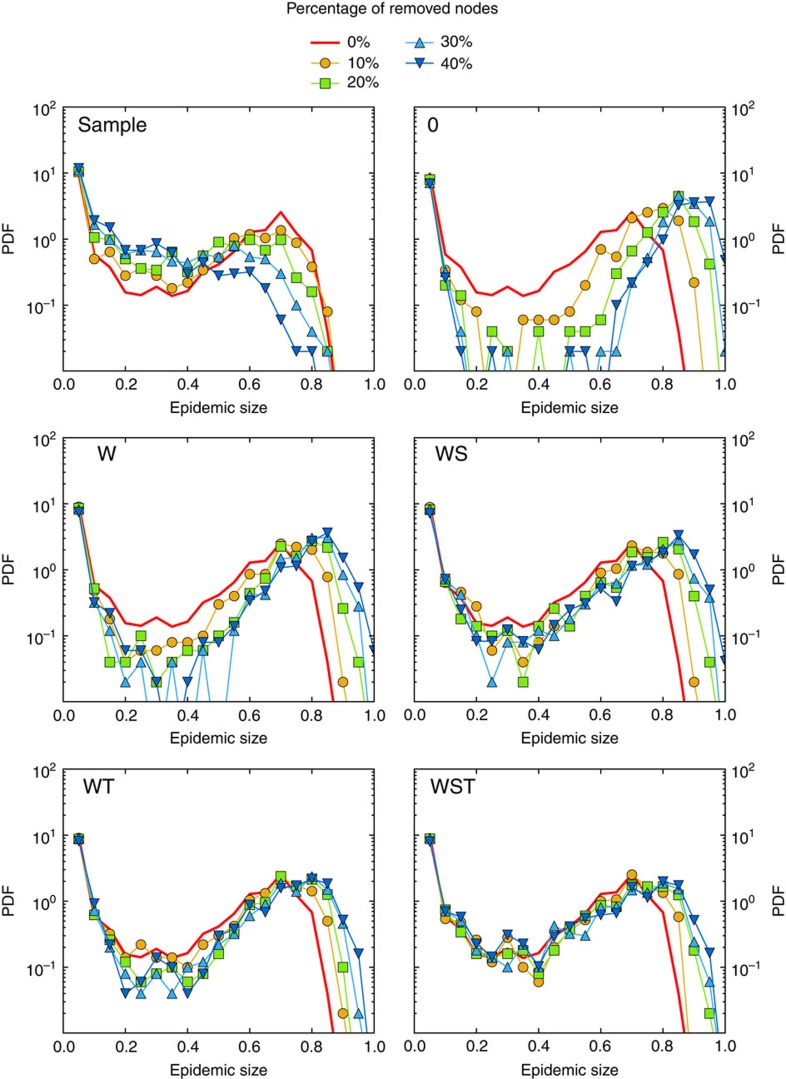
SIR simulations for the InVS workplace case. We compare of the outcome of SIR epidemic simulations performed on resampled and reconstructed contact networks, for different methods of reconstruction. We plot the distribution of epidemic sizes (fraction of recovered individuals) at the end of SIR processes simulated on top of resampled (sample) and reconstructed contact networks, for different values of the fraction *f* of nodes removed, and for the five reconstruction methods described in the text (0, W, WS, WT and WST). The parameters of the SIR models are *β*=0.0004 and *β*/*μ*=1,000. The case *f*=0 corresponds to simulations using the whole data set, that is, the reference case. For each value of *f*, 1,000 independent simulations were performed.

**Figure 3:**
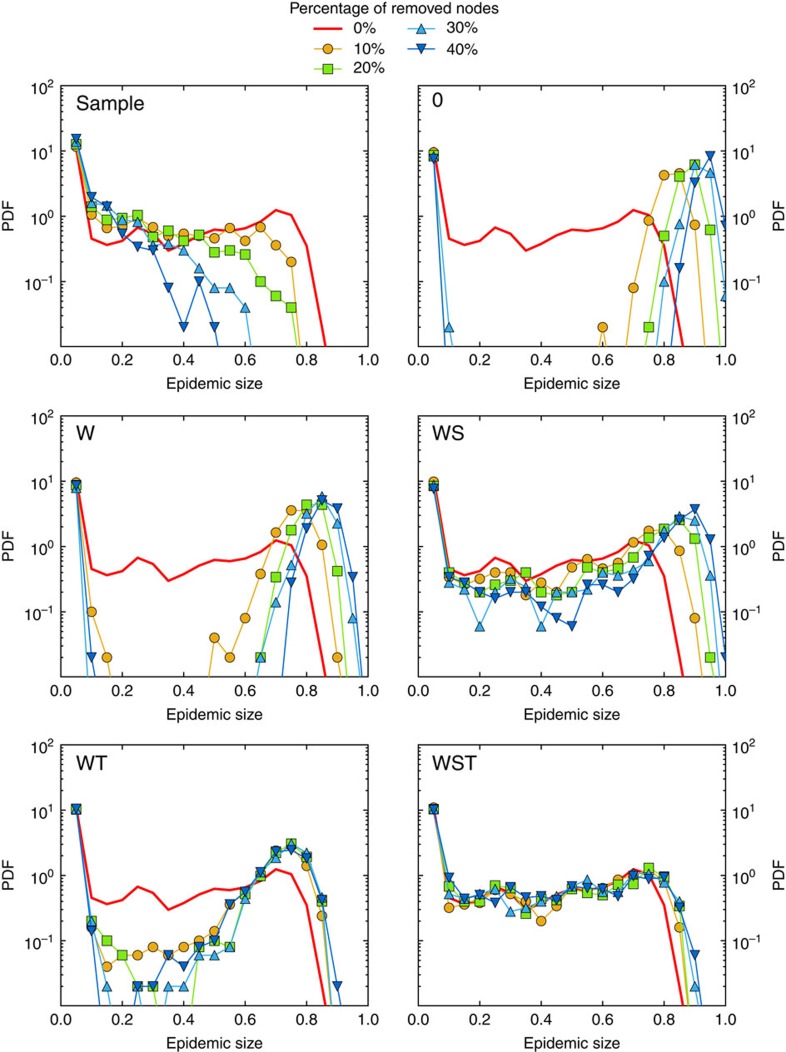
SIR simulations for the Thiers13 high school case. We compare of the outcome of SIR epidemic simulations performed on resampled and reconstructed contact networks, for different methods of reconstruction. We plot the distribution of epidemic sizes (fraction of recovered individuals) at the end of SIR processes simulated on top of resampled (sample) and reconstructed contact networks, for different values of the fraction *f* of nodes removed, and for the five reconstruction methods described in the text (0, W, WS, WT and WST). The parameters of the SIR models are *β*=0.0004 and *β*/*μ*=100. The case *f*=0 corresponds to simulations using the whole data set, that is, the reference case. For each value of *f*, 1,000 independent simulations were performed.

**Figure 4:**
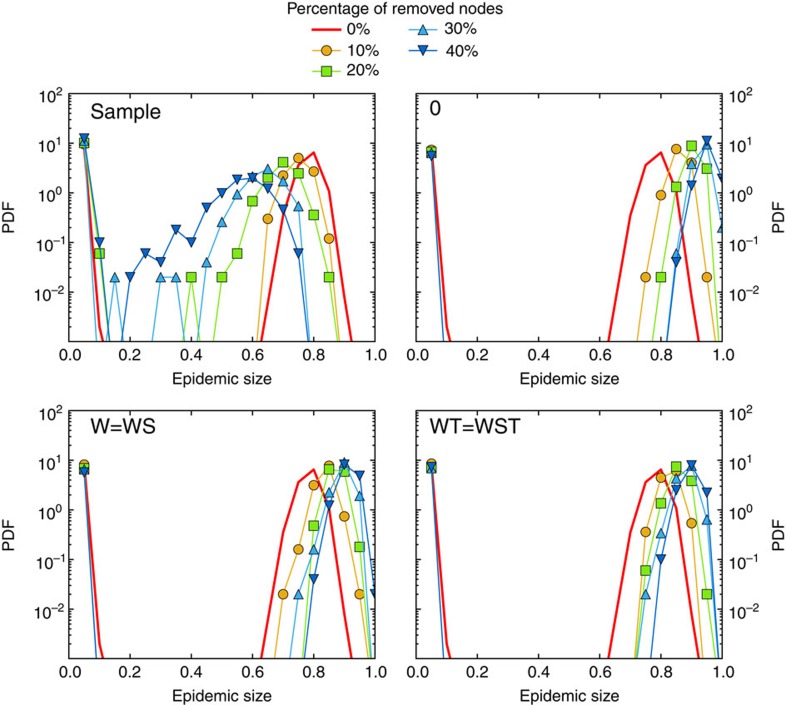
SIR simulations for the SFHH conference case. We compare of the outcome of SIR epidemic simulations performed on resampled and reconstructed contact networks, for different methods of reconstruction. We plot the distribution of epidemic sizes (fraction of recovered individuals) at the end of SIR processes simulated on top of resampled (sample) and reconstructed contact networks, for different values of the fraction *f* of nodes removed, and for three reconstruction methods described in the text (0, W and WT). In this case, as the population is not structured, methods W and WS on the one hand, WT and WST on the other hand, are equivalent. The parameters of the SIR models are *β*=0.0004 and *β*/*μ*=100. The case *f*=0 corresponds to simulations using the whole data set, that is, the reference case. For each value of *f*, 1,000 independent simulations were performed.

**Figure 5:**
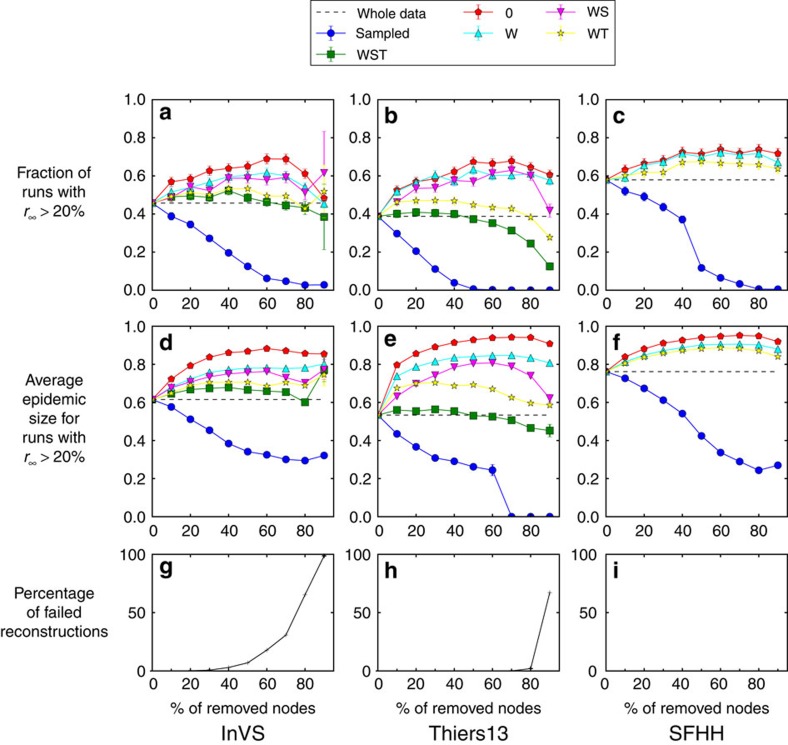
Accuracy of the different reconstruction methods. We perform SIR epidemic simulations for each case, for different values of the fraction *f* of missing nodes, for both sampled networks and networks reconstructed with the different methods. We compare in each case, and as a function of *f*, the fraction of outbreaks that lead to a final fraction of recovered individuals *r*_∞_ larger than 20% of the population (**a**–**c**) and the average size of these large outbreaks (**d**–**f**). The dashed lines give the corresponding values for simulations performed on the complete data sets. The different methods are: reconstruction conserving only the link density and the average weight of the resampled data (0); reconstruction conserving only the link density and the distribution of weights of the resampled data (W); reconstruction preserving, in addition to the W method, the group structure of the resampled data (WS); reconstruction conserving link density, distribution of weights and distributions of contact times, of inter-contact times and of numbers of contacts per link measured in the resampled data (WT); full method conserving all these properties (WST). We also plot as a function of *f* the failure rate of the WST algorithm, that is, the percentage of failed reconstructions (**g**–**i**). For the SFHH case, as the population is not structured into groups, methods W and WS are equivalent, as well as methods WT and WST; moreover, reconstruction is always possible. The SIR parameters are *β*=0.0004 and *β*/*μ*=1,000 (InVS) or *β*/*μ*=100 (Thiers13 and SFHH) and each point is averaged over 1,000 independent simulations.

Figures 2, 3, 4 display the outcome of SIR spreading simulations performed on surrogate networks obtained using the various reconstruction methods, compared with the outcome of simulations on the resampled data sets, for various values of *f*. Method 0 leads to a clear overestimation of the outcome and does not capture well the shape of the distribution of outbreak sizes. Method W gives only slightly better results. The overall shape of the distribution is better captured for the three reconstruction methods using more information: WS, WT and WST (note that for the SFHH case the population is not structured, so that W and WS are equivalent, as are WT and WST). The WST method matches best the shape of the distributions and yields distributions much more similar to those obtained by simulating on the whole data set than the simulations performed on the resampled networks. We also show in Fig. 5 the fraction of outbreaks that reach at least 20% of the population and the average epidemic size for these outbreaks. In the case of simulations performed on resampled data, we rapidly lose information about the size and even the existence of large outbreaks as *f* increases. Simulations using data reconstructed with methods 0 and W, on the contrary, largely overestimate these quantities, which is expected as infections spread easier on random graphs than on structured graphs^50^,^52^, especially if the heterogeneity of the aggregated contact durations is not considered^22^,^20^. Taking into account the population structure or using contact sequences that respect the temporal heterogeneities (broad distributions of contact and inter-contact durations) yield better results (WS and WT cases, respectively). Overall, the WST method, for which the surrogate networks respect all these constraints, yields the best results.

**Figure 6:**
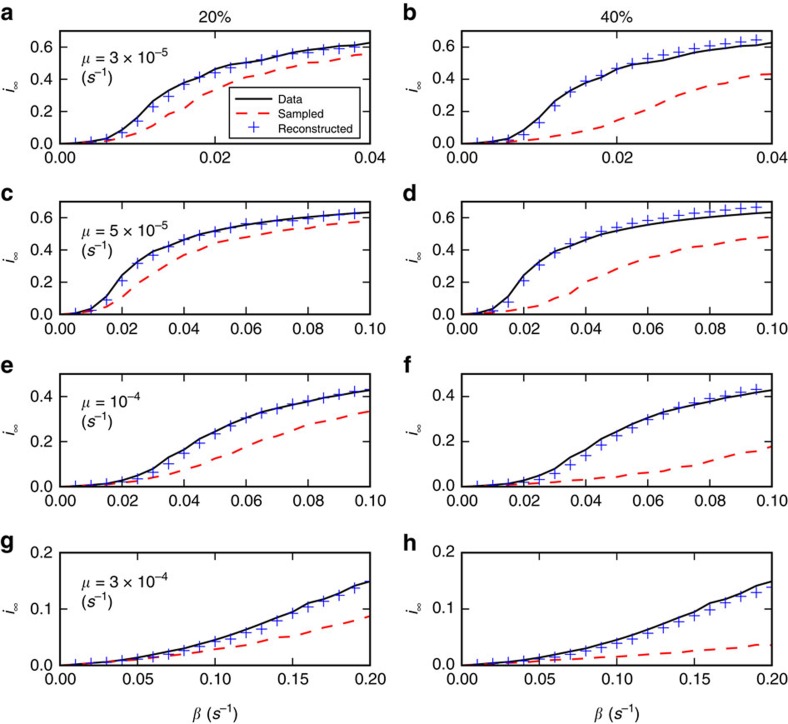
SIS simulations for the InVS workplace case. We perform SIS epidemic simulations and report the phase diagram of the SIS model for the original, resampled and reconstructed contact networks. Each panel shows the stationary value *i*_∞_ of the prevalence in the stationary state of the SIS model, computed as described in Methods, as a function of *β*, for several values of *μ*. Simulations are performed in each case using either the complete data set (continuous lines), resampled data (dashed lines) or reconstructed contact networks using the WST method (pluses). The fraction of excluded nodes in the resampling is *f*=20% for **a**,**c**,**e**,**g** and *f*=40% for **b**,**d**,**f**,**h**.

We show in the Supplementary Figs 15–18 that similar results are obtained for different values of the spreading parameters. Moreover, the phase diagram obtained for the SIS model when using reconstructed networks is much closer to the original than for resampled networks (Fig. 6 and Supplementary Figs 19–20). Overall, simulations on networks reconstructed using the WST method yield a much better estimation of the epidemic risk than simulations using resampled network data, for both SIS and SIR models.

### Reshuffled data sets

Even when simulations are performed on reconstructed contact patterns built with the WST method, the maximal outbreak sizes are systematically overestimated (Figs 2, 3, 4), as well as, in most cases, the probability and average size of large outbreaks, especially for SFHH (Figs 4, 5). These discrepancies might stem from structural and/or temporal correlations present in empirical contact data that are not taken into account in our reconstruction methods. To test this hypothesis, we construct several reshuffled data sets and use them as initial data in our resampling and reconstruction procedure. We use both structural and temporal reshuffling as described in the Methods section, in order to remove either structural correlations, temporal correlations, or both, from the original data sets. We then proceed to a resampling and reconstruction procedure (using the WST method) as for the original data, and perform numerical simulations of SIR processes. As for the original data, simulations on resampled data lead to a strong underestimation of the process outcome, and simulations using the reconstructed data gives much better results.

**Figure 7:**
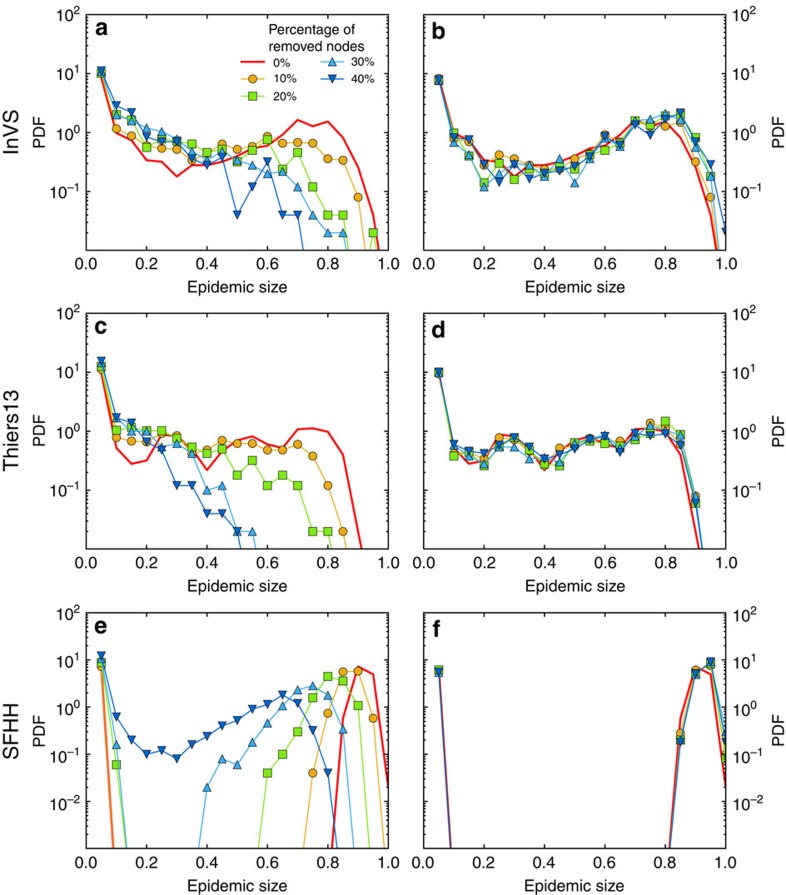
SIR simulations on shuffled data. We compare of the outcome of SIR epidemic simulations performed on resampled and reconstructed contact networks, for shuffled data. We plot the distribution of epidemic sizes (fraction of recovered individuals) at the end of SIR processes simulated on top of either resampled (**a**,**c**,**e**) or reconstructed (**b**,**d**,**f**) contact networks, for different values of the fraction *f* of nodes removed. We use here the WST reconstruction method, and the data set considered consists in a CM-shuffled version (see Methods) of the original data, in which the shuffling procedure removes structural correlations of the contact network within each group. The parameters of the SIR models are *β*=0.0004 and *β*/*μ*=1,000 (InVS) or *β*/*μ*=100 (Thiers13 and SFHH). The case *f*=0 corresponds to simulations using the whole data set, that is, the reference (reshuffled data) case. For each value of *f*, 1,000 independent simulations were performed.

We show in the Supplementary Figs 21–22 that we still obtain discrepancies, and in particular overestimations of the largest epidemic sizes, when we use temporally reshuffled data in which the link structure of the contact network is maintained. If on the other hand we use data in which the network structure has been reshuffled in a way to cancel structural correlations within each group, the reconstruction procedure gives a very good agreement between the distributions of epidemic sizes of original and reconstructed data, as shown in Fig. 7. More precisely we consider here ‘CM-shuffled' data, that is, contact networks in which the links have been reshuffled randomly but separately for each pair of groups, that is, a link between an individual of group A and an individual of group B is still between groups A and B in the reshuffled network. The difference with the case of non-reshuffled empirical data are particularly clear for the SFHH case. This indicates that the overestimation observed in Figs 2, 3, 4 is mostly due to the fact that the reconstructed data does not reproduce small scale structures of the contact networks: such structures might be owing to for example, groups of colleagues or friends, whose composition is neither available as metadata nor detectable in the resampled data sets.

### Limitations

**Figure 8:**
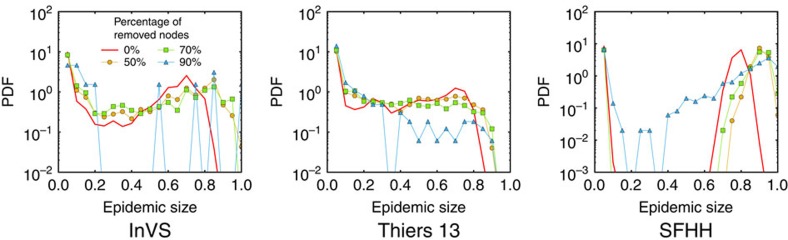
SIR simulations for large fractions of missing nodes. We simulate SIR processes on reconstructed contact networks for large values of the fraction *f* of removed nodes. We plot the distributions of epidemic sizes for simulations on reconstructed networks and on the whole data set (case *f*=0), for large values of the fraction *f* of removed nodes. Here *β*=0.0004 and *β*/*μ*=1,000 (InVS) or *β*/*μ*=100 (Thiers13 and SFHH) and 1,000 simulations were performed for each value of *f*. The distributions of epidemic sizes for simulations performed on resampled data sets are not shown since at these high values of *f*, almost no epidemics occur.

When the fraction *f* of nodes excluded by the resampling procedure becomes large, the properties of the resampled data may start to differ substantially from those of the whole data set (Supplementary Figs 1–2). As a result, the distributions of epidemic sizes of SIR simulations show stronger deviations from those obtained on the whole data set (Fig. 8), even if the epidemic risk evaluation is still better than for simulations on the resampled networks (Fig. 5). Most importantly, the information remaining in the resampled data at large *f* can be insufficient to construct surrogate contacts. This happens in particular if an entire class or department is absent from the resampled data or if all the resampled nodes of a class/department are disconnected (see Methods for details). We show in the bottom plots of Fig. 5 the failure rate, that is, the fraction of cases in which we are not able to construct surrogate networks from the resampled data. It increases gradually with *f* for the InVS data since the groups (departments) are of different sizes. For the Thiers13 data, all classes are of similar sizes so that the failure rate reaches abruptly a large value at a given value of *f*. For the SFHH data, we can always construct surrogate networks as the population is not structured. Another limitation of the reconstruction method lies in the need to know the number of individuals missing in each department or class. If these numbers are completely unknown, giving an estimation of outbreak sizes is impossible as adding arbitrary numbers of nodes and links to the resampled data can lead to arbitrarily large epidemics. The methods are, however, still usable if only partial information is available. For instance, if only the overall missing number of individuals is available, it is possible to use the WT method, which still gives sensible results. Moreover, if *f* is only approximately known, for example, *f* is known to be within an interval of possible values (*f*_1_, *f*_2_), it is possible to perform reconstructions using the respective hypothesis *f*=*f*_1_ and *f*=*f*_2_ and to give an interval of estimates. We provide an example of such procedure in Supplementary Fig. 23.

## Discussion

The understanding of epidemic spreading phenomena has been vastly improved thanks to the use of data-driven models at different scales. High-resolution contact data in particular have been used to evaluate epidemic risk or containment policies in specific populations or to perform contact tracing^14^,^19^,^20^,^28^,^29^,^31^,^32^. In such studies, missing data due to population sampling might represent, however, a serious issue: individuals absent from a data set are equivalent to immunised individuals when epidemic processes are simulated. Feeding sampled data into data-driven models can therefore lead to severe underestimations of the epidemic risk and might even affect the evaluation of mitigation strategies if for instance some at-risk groups are particularly undersampled.

Here we have put forward a set of methods to obtain a better evaluation of the outcome of spreading simulations for data-driven models using contact data from a uniformly sampled population. To this aim, we have shown how it is possible, starting from a data set describing the contacts of only a fraction of the population of interest (uniformly sampled from the whole population), to construct surrogate data sets using various amounts of accessible information, that is, quantities measured in the sampled data. We have shown that the simplest method, which consists in simply compensating for the decrease in the average number of neighbours due to sampling, yields a strong overestimation of the epidemic risk. When additional information describing the group structure and the temporal properties of the data is added in the construction of surrogate data sets, simulations of epidemic spreading on such surrogate data yield results similar to those obtained on the complete data set. We note here that the issue of how much information should be included when constructing the surrogate data is linked to the general issue of how much information is needed to get an accurate picture of spreading processes on temporal networks^22^,^27^,^28^,^29^,^53^,^54^. Some discrepancies in the epidemic risk estimation are, however, still observed, due in particular to small scale structural correlations of the contact network that are difficult or even impossible to measure in the resampled data: these discrepancies are indeed largely suppressed if we use as original data a reshuffled contact network in which such correlations are absent.

The methods presented here yield much better results than simulations using resampled data, even when a substantial part of the population is excluded, in particular in estimating the probability of large outbreaks. It suffers, however, from limitations, especially when the fraction *f* of excluded individuals is too large. First, the construction of the surrogate contacts relies on the stability of a set of quantities with respect to resampling, but the measured quantities start to deviate from the original ones at large *f*. The shape of the distribution of epidemic sizes may then differ substantially from the original one. Second, large values of *f* might even render the construction of the surrogate data impossible due to the loss of information on whole categories of nodes. Finally, at least an estimate of the number of missing individuals in the population is needed in order to create a surrogate data set.

An interesting avenue for future work concerns possible improvements of the reconstruction methods, in particular by integrating into the surrogate data additional information and complex correlation patterns measured in the sampled data. For instance, the number of contacts varies significantly with the time of day in most contexts: the corresponding activity timeline might be measured in the sampled data (overall or even for each group of individuals), assumed to be robust to sampling and used in the reconstruction of contact timelines. More systematically, it might also be possible to use the temporal network decomposition technique put forward in ref. 55, on the sampled data, to extract mesostructures such as temporally localized mixing patterns. The surrogate contacts could then be built in a way to preserve such patterns. Indeed, correlations between structure and activity in the temporal contact network are known to influence spreading processes^21^,^52^,^54^,^56^,^57^,^58^ but are notoriously difficult to measure. If the group structure of the population is unknown, recent approaches based on stochastic block models^59^ might be used to extract groups from the resampled data; this extracted group structure could then be used to build the corresponding contact matrix and surrogate data sets.

We finally recall that we have assumed an uniform sampling of nodes, corresponding to an independent random choice of each individual of the population to take part or not to the data collection. Other types of sampling or data losses can, however, also be present in data collected by wearable sensors, such as partial coverage of the premises of interest by the measuring infrastructure, non-uniform sampling depending on individual activity (too busy persons or, on the contrary, asocial individuals, might not want to wear sensors), on group membership, or due to clusters of non-participating individuals (for example, groups of friends). In addition, other types of data sets such as the ones obtained from surveys or diaries correspond to different types of sampling, as each respondent provides then information in the form of an ego network^60^. Such data sets involve potentially additional types of biases such as underreporting of the number of contacts and overestimation of contact durations^8^,^9^,^61^: how to adapt the methods presented here is an important issue that we will examine in future work. Finally, the population under study is (usually) not isolated from the external world, and it would be important to devise ways to include contacts with outsiders in the data and simulations, for instance by using other data sources such as surveys.

## Methods

### Data

We consider data sets collected using the SocioPatterns proximity sensing platform (http://www.sociopatterns.org) based on wearable sensors that detect close face-to-face proximity of individuals wearing them. Informed consent was obtained from all participants and the French national bodies responsible for ethics and privacy, the Commission Nationale de l′Informatique et des Libertés (http://www.cnil.fr), approved the data collections.

The high school (Thiers13) data set^61^ is structured in nine classes, forming three subgroups of three classes corresponding to their specialisation in Mathematics-Physics (MP, MP*1, MP*2 with respectively 31, 29 and 38 students), Physics (PC, PC*, PSI with respectively 44, 39 and 34 students), or Biology (2BIO1, 2BIO2 and 2BIO3 with respectively 37, 35 and 39 students).

The workplace (InVS) data set^46^ is structured in five departments: DISQ (Scientific Direction, 15 persons), DMCT (Department of Chronic Diseases and Traumatisms, 26 persons), DSE (Department of Health and Environment, 34 persons), SRH (Human Resources, 13 persons) and SFLE (Logistics, four persons).

For the conference data (SFHH), we do not have metadata on the participants, and the aggregated network structure was found to be homogeneous^22^.

### SIR and SIS simulations

Simulations of SIR and SIS processes on the temporal networks of contacts (original, resampled or reconstructed) are performed using the temporal Gillespie algorithm described in ref. 62. For each run of the simulations, all nodes are initially susceptible; a node is chosen at random as the seed of the epidemic and put in the infectious state at a point in time chosen at random over the duration of the contact data. A susceptible node in contact with an infectious node becomes infectious at rate *β*. Infectious nodes recover at rate *μ*: in the SIR model they then enter the recovered state and cannot become infectious again, while in the SIS model they enter the susceptible state again. If needed, the sequence of contacts is repeated in the simulation^22^.

For SIR processes, we run each simulation, with the seed node chosen at random, until no infectious individual remains (nodes are thus either still susceptible or have been infected and then recovered). We consider values of *β* and *μ* yielding a non-negligible epidemic risk, that is, such that a rather large fraction of simulations lead to a final size larger than 20% of the population (Figs 1, 2, 3, 4): **β**=4 × 10^−4^ s^−1^, **μ**=4 × 10^−7^ s^−1^ (InVS) or 4 × 10^−6^ s^−1^ (SFHH and Thiers13). Other parameter values are explored in the Supplementary Figs 15–18. For each set of parameters, the distribution of epidemic sizes is obtained by performing 1,000 simulations.

For SIS processes, simulations are performed using the quasi-stationary approach of ref. 63. They are run until the system enters a stationary state as witnessed by the mean number of infected nodes being constant over time. Simulations are then continued for 50,000 time-steps while recording the number of infected nodes. For each set of parameters, the simulations are performed once with each node of the network chosen as the seed node.

### Reconstruction algorithm

We consider a population 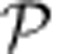 of *N* individuals, potentially organised in groups. We assume that all the contacts occurring among a subpopulation 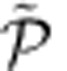 of these individuals, of size 
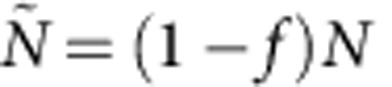
, are known. This constitutes our resampled data from which we need to construct a surrogate set of contacts concerning the remaining 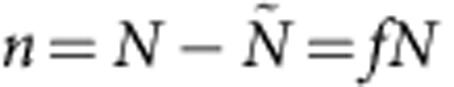 individuals for which no contact information is available: these contacts can occur among these individuals and between them and the members of 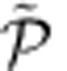. We assume that we know the group of each member of 
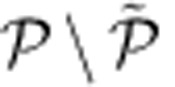
, and the overall activity timeline, that is, the intervals during which contacts take place, separated by nights and weekends.

To construct the surrogate data (WST method), we first compute from the activity timeline the total duration *T*_u_ of the periods during which contacts can occur.

Then, we measure in the sampled data:

- The density *ρ* of links in the aggregated contact network;
- A row-normalised contact matrix *C*, in which the element *C*_AB_ gives the probability for a node of group A to have a link to a node of group B;
- The list {*τ*_c_} of contact durations;
- The lists {*τ*_i*c*_}^int^ and {*τ*_i*c*_}^ext^ of inter-contact durations for internal and external links, that is, for links between nodes of the same group and links between nodes that belong to two different groups, respectively;
- The lists {*p*}^int^ and {*p*}^ext^ of numbers of contacts per link, respectively for internal (within groups) and external (between groups) links;
- The list {*t*_0_} of initial times between the start of the data set and the first contact between two nodes.

Given *ρ*, we compute the number *e* of additional links needed to keep the network density constant when we add the *n* excluded nodes. We then construct each link according to the following procedure:

- A node *i* is randomly chosen from the set 
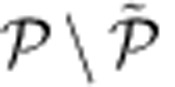
 of excluded nodes;
- Knowing the group A that *i* belongs to, we extract at random a target group B with probability given by *C*_AB_;
- We draw a target node *j* at random from B (if B=A, we take care that *i*≠*j*) such that *i* and *j* are not linked;
- Depending on whether *i* and *j* belong to the same group or not, we draw from {*p*}^int^ or {*p*}^ext^ the number of contact events *p* taking place over the link *ij*;
- From {*t*_0_}, we draw the initial waiting time before the first contact;
- From {*τ*_c_}, we draw *p* contact durations 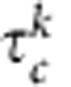 , *k*=1,⋯,*p*;
- From {*τ*_i*c*_}^int^ or {*τ*_i*c*_}^ext^, we draw *p*−1 inter-contact durations 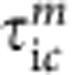 , *m*=1,⋯,*p* − 1;
- If 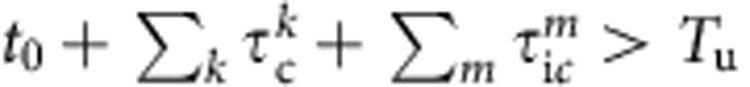 , we repeat the drawing of temporal characteristics until we obtain a set of values such that 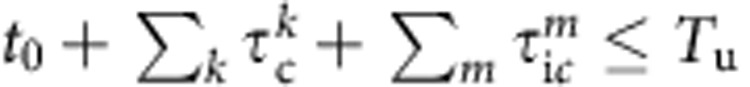 ;
- From *t*_0_ and the 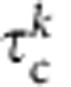 and 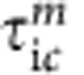 , we build the contact timeline of the link *ij*;
- Finally, we insert in the contact timeline the breaks defined by the global activity timeline.

### Possible failure of the reconstruction method at large *f*

The construction of the surrogate version of the missing links uses as an input the group structure of the subgraph that remains after sampling, as given by the contact matrix of the link densities between the different groups of nodes that are present in the subpopulation 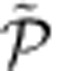. Depending on the characteristics of 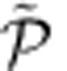 and of the corresponding contacts, the construction method can fail in several cases: (i) if an entire group (class/department) of nodes in the population is absent from 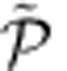; (ii) if the remaining nodes of a specific group (class/department) are all isolated in 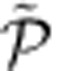
's contact network; (iii) if, during the algorithm, a node of 
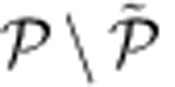
 is selected in a certain group A but cannot create any more links because it already has links to all nodes in the groups B such that *C*_AB_≠0; (iv) if there are either no internal (within groups) or external (between groups) links in the contact network of 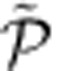: in this case one of the lists of link temporal characteristics is empty and the corresponding structures cannot be reconstructed.

Cases (i) and (ii) correspond to a complete loss of information about the connectivity of a group (class/department) of the population, due to sampling. It is then impossible to reconstruct a sensible connectivity pattern for these nodes. Case (iii) is more subtle and occurs in situations of very low connectivity between groups. For instance, within the contact network of 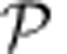, a group A has links only with another specific group B, and both A and B are small; it is then possible that the nodes of 
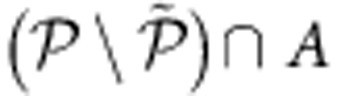
 exhaust the set of possible links to nodes of B during the reconstruction algorithm. If a node of 
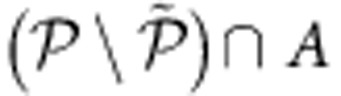
 is again chosen to create a link, such a creation is not possible and the construction fails. Case (iv) usually corresponds to situations in which the links between individuals of different groups which remain in the resampled data set correspond to pairs of individuals who have had only one contact event: in such cases, {*τ*_i*c*_}^ext^ is empty and external links with more than one contact cannot be built.

### Shufflings

To test the effect of correlations in the temporal network, we use four shuffling methods, based on the ones defined in ref. 56.

Link shuffling. The contact timelines associated with each link are randomly redistributed among the links. Correlations between timelines of links adjacent to a given node are destroyed, as well as correlations between weights and topology. The structure of the network is kept, as well as the global activity timeline.

Time shuffling. From the contact data we build the lists {*τ*_c_}, {*τ*_i*c*_} and {*p*} of, respectively, contact durations, inter-contact durations and number of contacts per link. We also measure the list {*t*_0_} of initial times between the start of the data set and the first contact between two nodes. For each link, we draw randomly a starting time *t*_0_, a number *p* of contacts from {*p*}, *p* contact durations from {*τ*_c_} and *p*−1 inter-contact durations from {*τ*_i*c*_}, so that the total duration of the timeline does not exceed the total available time *T*_u_. We then construct the contact timelines, thus destroying the temporal correlations among contacts. The structure of the network is instead kept fixed.

CM shuffling. We perform a link rewiring separately on each compartment of the contact matrix, that is, we randomly redistribute links with their contact timelines within each group, and within each pair of groups. We thus destroy the structural correlations inside each compartment of the contact matrix, while preserving the group structure of the network as given by the link density contact matrix and the contact matrix of total contact times between groups.

CM-time shuffling. We perform both a CM shuffling and a time shuffling.

## Additional information

**How to cite this article:** Génois, M. *et al.* Compensating for population sampling in simulations of epidemic spread on temporal contact networks. *Nat. Commun.* 6:8860 doi: 10.1038/ncomms9860 (2015).

## Supplementary Material

Supplementary Information Supplementary Figures 1-23, Supplementary Notes 1-2 and Supplementary Methods,

## Figures and Tables

**Table 1:** **Data sets.**

**Data set**	**Type**	***N***	***r***	***T***	**Dates**
InVS	Workplace	92	63%	2 weeks	24 June–5 July 2013
Thiers13	High school	326	86%	1 week	2–7 December 2013
SFHH	Conference	403	34%	2 days	3–4 June 2009

For each data set we specify the type of social situation, the number *N* of individuals whose contacts were measured, the corresponding participation rate *r*, the duration *T* and the dates of the data collection.

**Table 2:** Contact matrix similarities.

	***f***	**InVS CML**	**Thiers13 CML**
	10%	0.996 (0.937,0.999)	0.999 (0.998,0.999)
Resampled	20%	0.980 (0.889,0.994)	0.996 (0.995,0.997)
	40%	0.925 (0.872,0.983)	0.988 (0.983,0.990)
	10%	0.976 (0.846,0.995)	0.998 (0.994,0.999)
Reconstructed	20%	0.942 (0.844,0.984)	0.993 (0.985,0.995)
	40%	0.890 (0.652,0.953)	0.977 (0.938,0.987)

Similarities between the original contact matrices and the contact matrices of the resampled networks (top) and of the reconstructed networks (bottom). Median and 90% confidence interval for the cosine similarity between link density contact matrices (CML) for different values of *f*, the fraction of nodes removed from the original data. Values were obtained from 100 independent realisations of the resampling and reconstruction procedures.
