# Supplementary material for: Compensating for population sampling in simulations of epidemic spread on temporal contact networks
Source: Nat Commun. 2015 Nov 13;6:8860. doi: 10.1038/ncomms9860 (PMC4660211; doi:10.1038/ncomms9860)
Supplement: Supplementary Information — Supplementary Figures 1-23, Supplementary Notes 1-2 and Supplementary Methods, [file ncomms9860-s1.pdf]

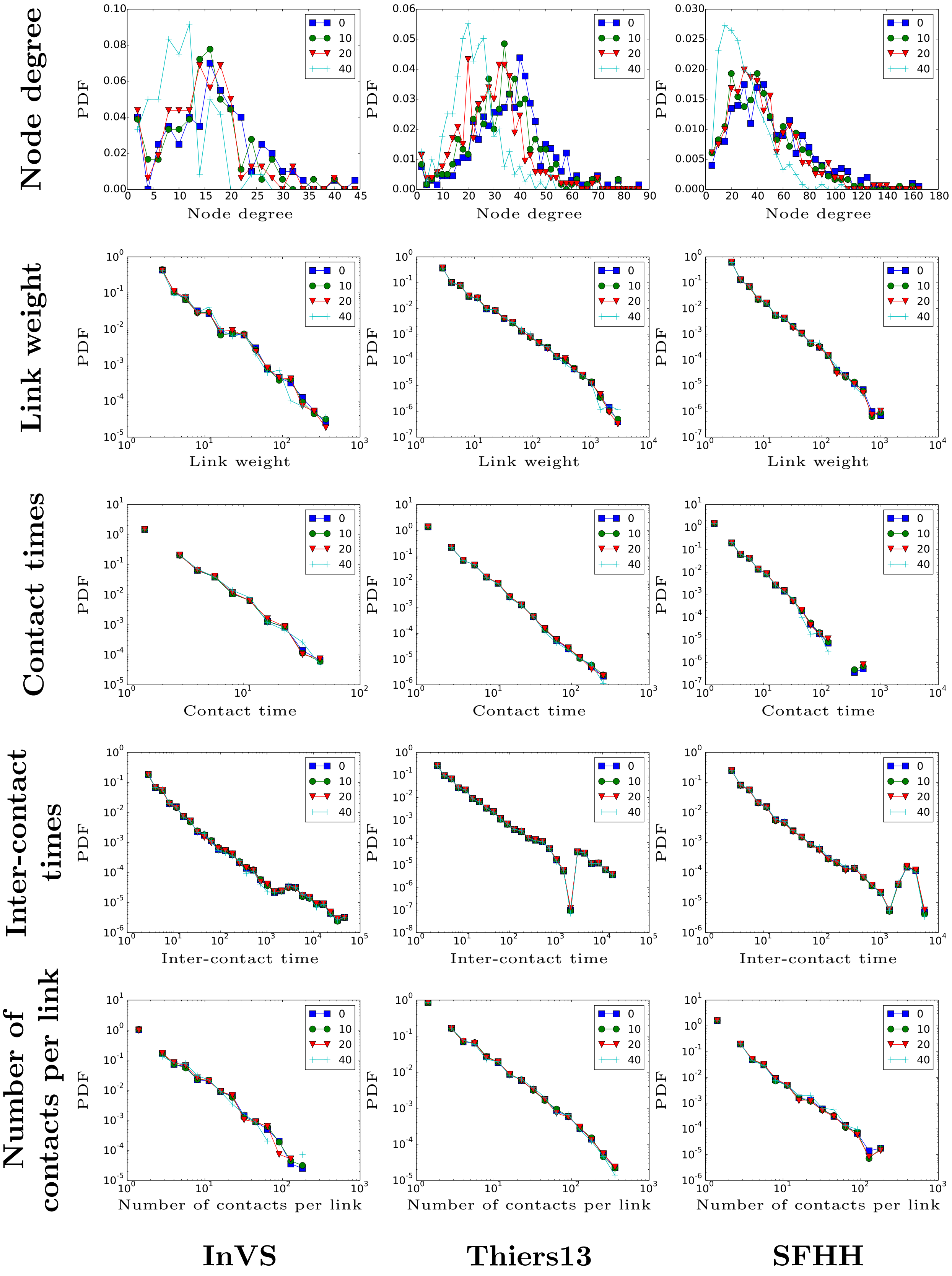

Supplementary Fig. 1. Effect of sampling on contact network properties. Comparison of the distributions of structural (node degrees and link weights in the aggregated network of contacts) and temporal (contact durations, inter-contact times, number of contacts per link) properties of the contact networks, for different fractions f of removed nodes. For each value of f, the distributions are computed on a single realisation of the resampling.

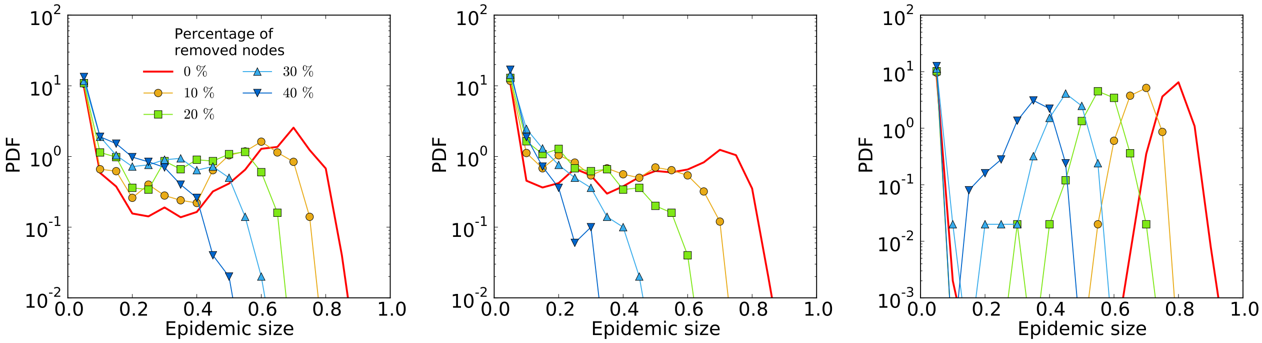

InVS **Thiers13** **SFHH**

Supplementary Fig. 2. **Effect of sampling on network density and on the similarity of contact matrices.** (Left) Density ρ of the aggregated network of contacts as a function of the fraction f of nodes excluded. The shaded areas represent mean $\rho \pm$ s.e.m.. (Right) Median cosine similarities between the link density contact matrices (CML) of resampled and full data sets, as a function of f, for the structured populations (high school and workplace). Results are averaged, for each value of f, over $1,000$ realisations for the density and over 100 realisations for the similarities.

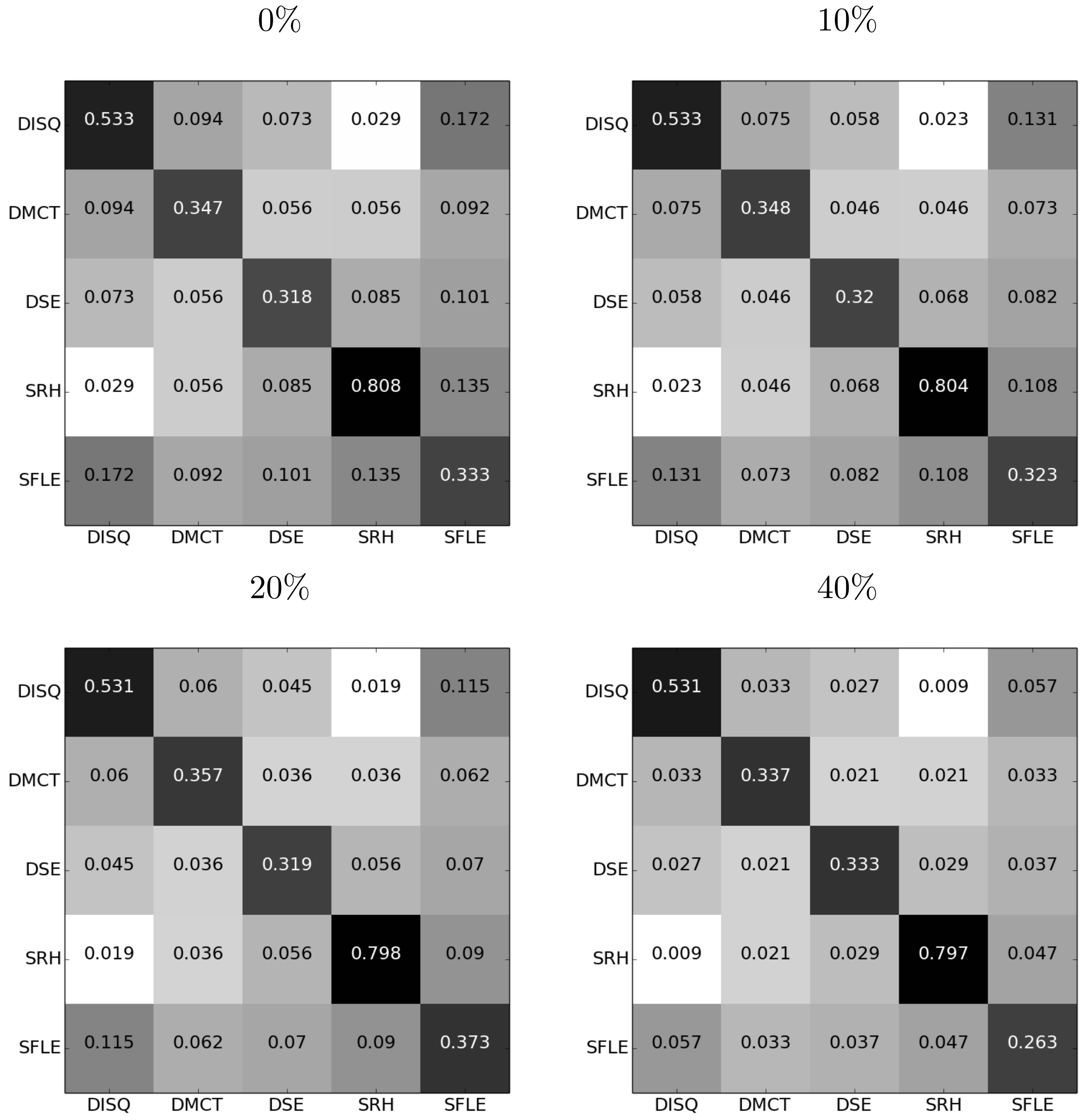

Supplementary Fig. 3. **Effect of sampling: link density contact matrices (*InVS*).** Comparison of link density contact matrices for the workplace, for different fractions of excluded nodes, f, with the original one ($f = 0$). Each matrix element AB gives the number of links between nodes of department A and nodes of department B in the contact network, normalised by the maximum possible number of such links. For each value of f, each matrix element is an average over 100 realisations of the sampling.

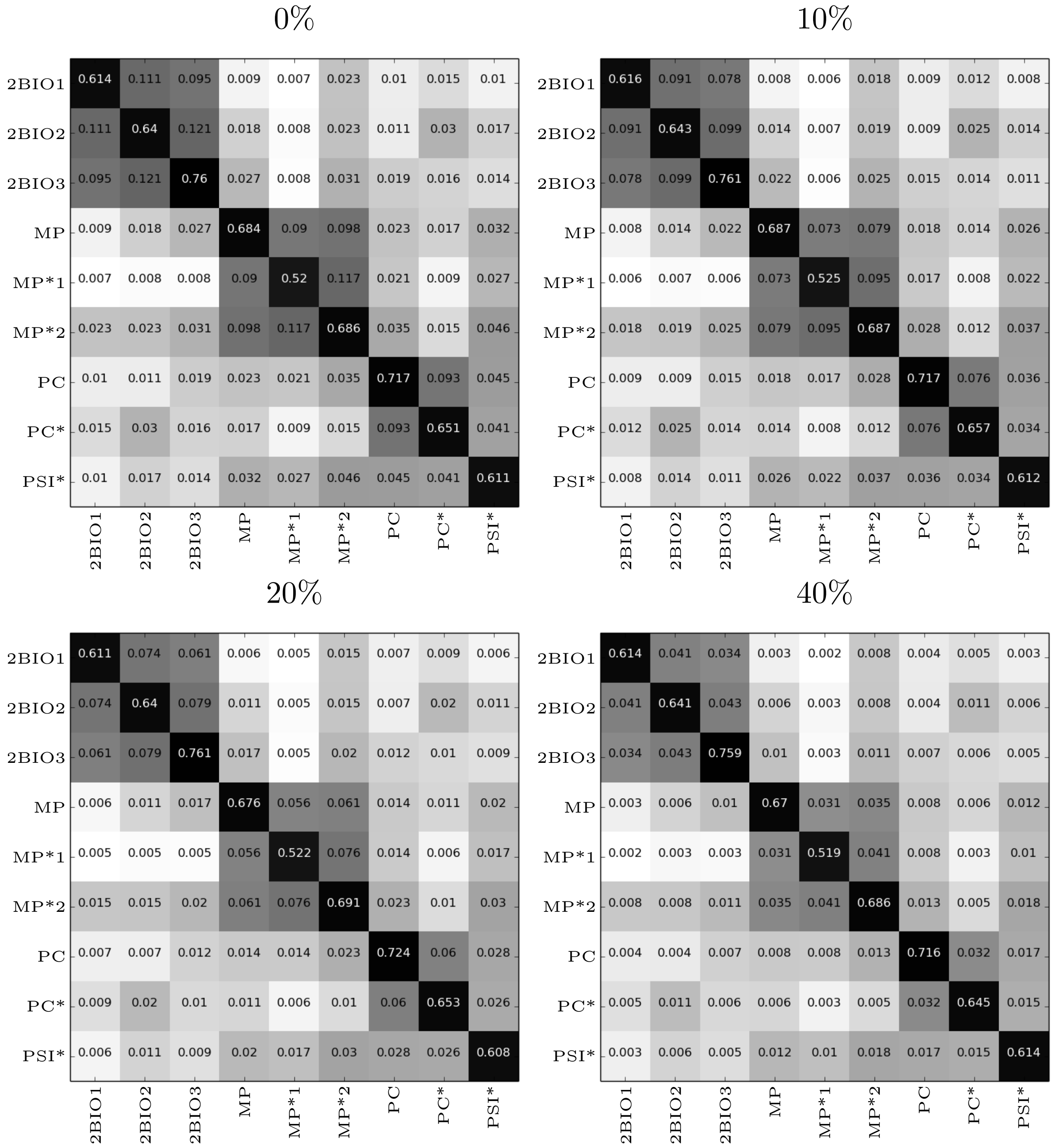

Supplementary Fig. 4. Effect of sampling: link density contact matrices (*Thiers13*). Comparison of the link density contact matrices for the high school, for different fractions f of excluded nodes, with the original one ($f = 0$). Each matrix element AB gives the number of links between nodes of class A and nodes of class B in the contact network, normalised by the maximum possible number of such links. For each value of f, each matrix element is an average over 100 realisations of the sampling.

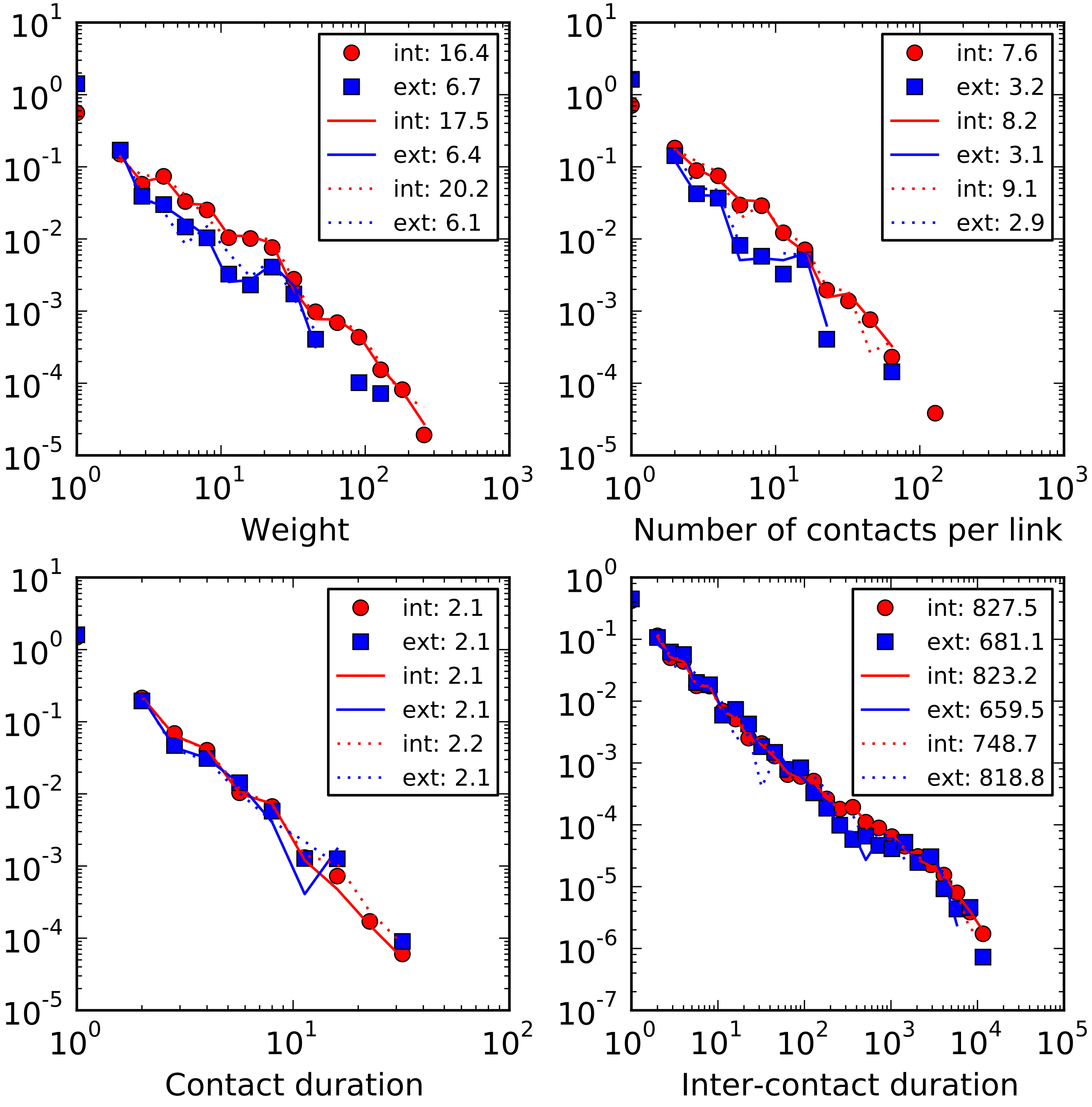

Supplementary Fig. 5. Distributions of temporal characteristics for internal (within groups) and external (between groups) contacts and links (*InVS* data). Symbols are for the original data, full lines for resampled data with $f = 20\%$, dotted lines for $f = 40\%$. Legends give the average values for each distribution.

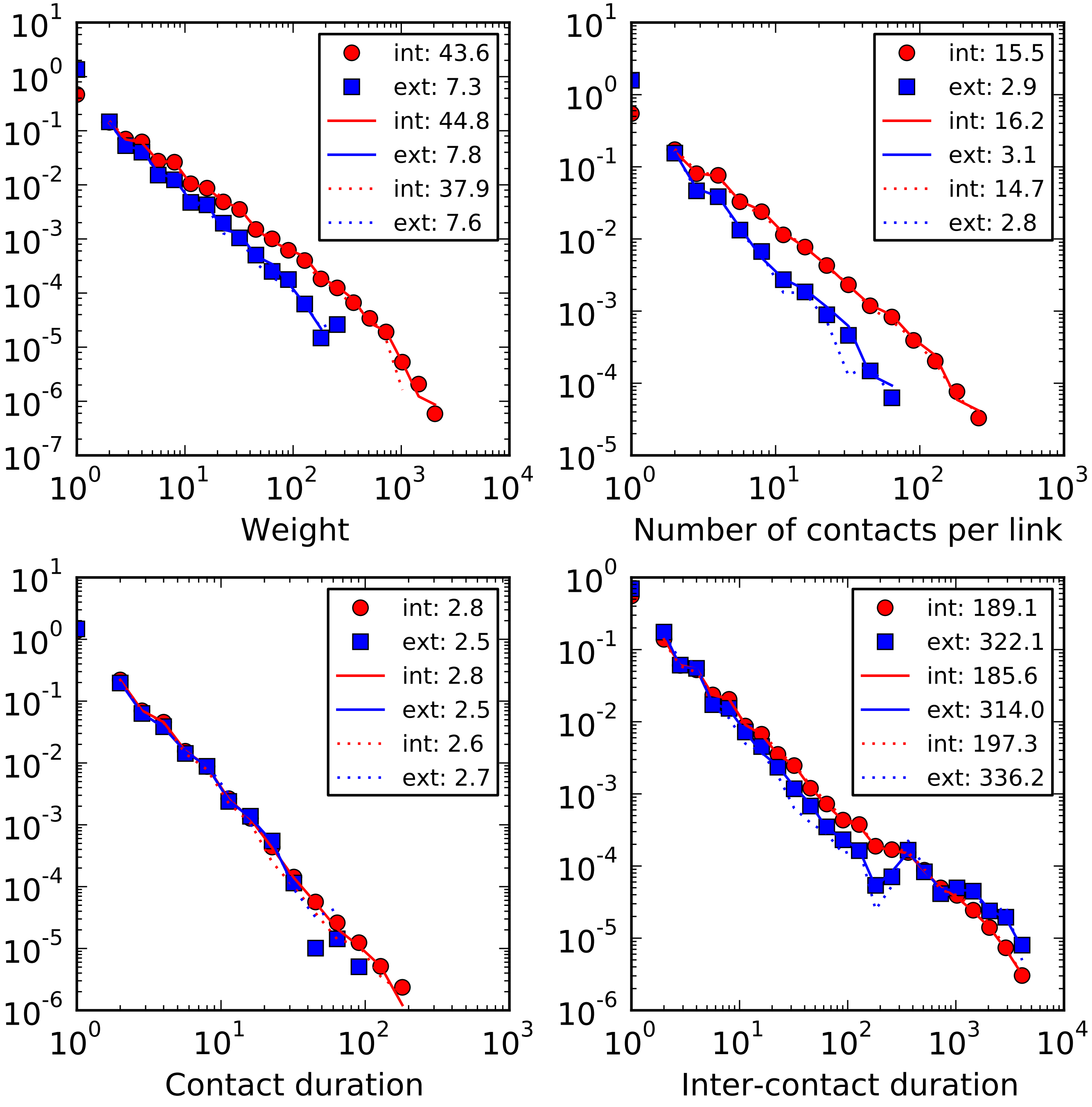

Supplementary Fig. 6. Distributions of temporal characteristics for internal (within groups) and external (between groups) contacts and links (*Thiers13* data). Symbols are for the original data, full lines for resampled data with $f = 20\%$, dotted lines for $f = 40\%$. Legends give the average values for each distribution.

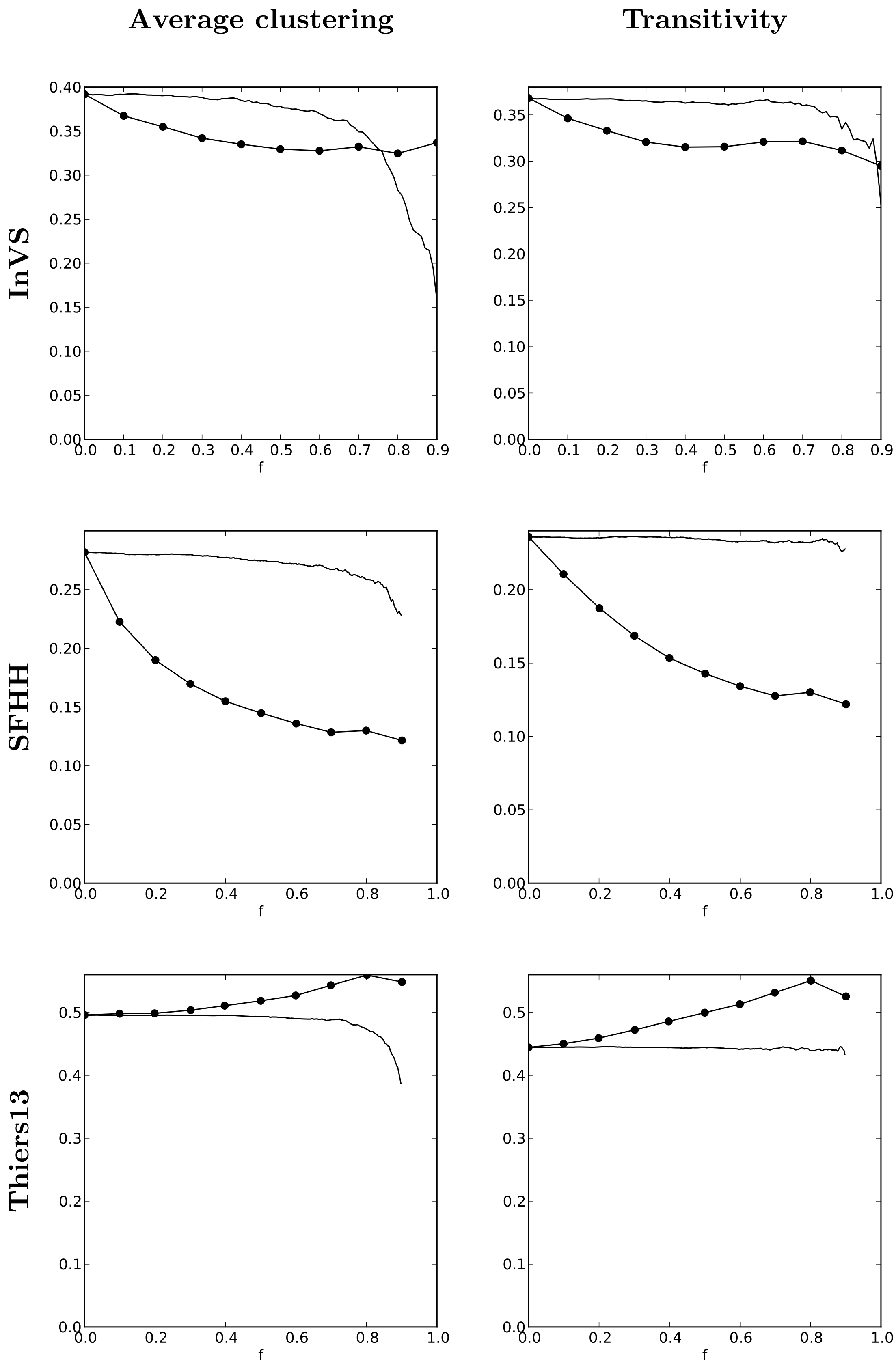

Supplementary Fig. 7. Effect of sampling and reconstruction on the average clustering coefficient (left column) and on the network transitivity (right column). The continuous lines show the evolution of clustering coefficient (left) and network transitivity when the fraction f of removed nodes increases. The full circles correspond to the same quantities for networks reconstructed using the **WST** method. Each point is averaged on 100 realisations.

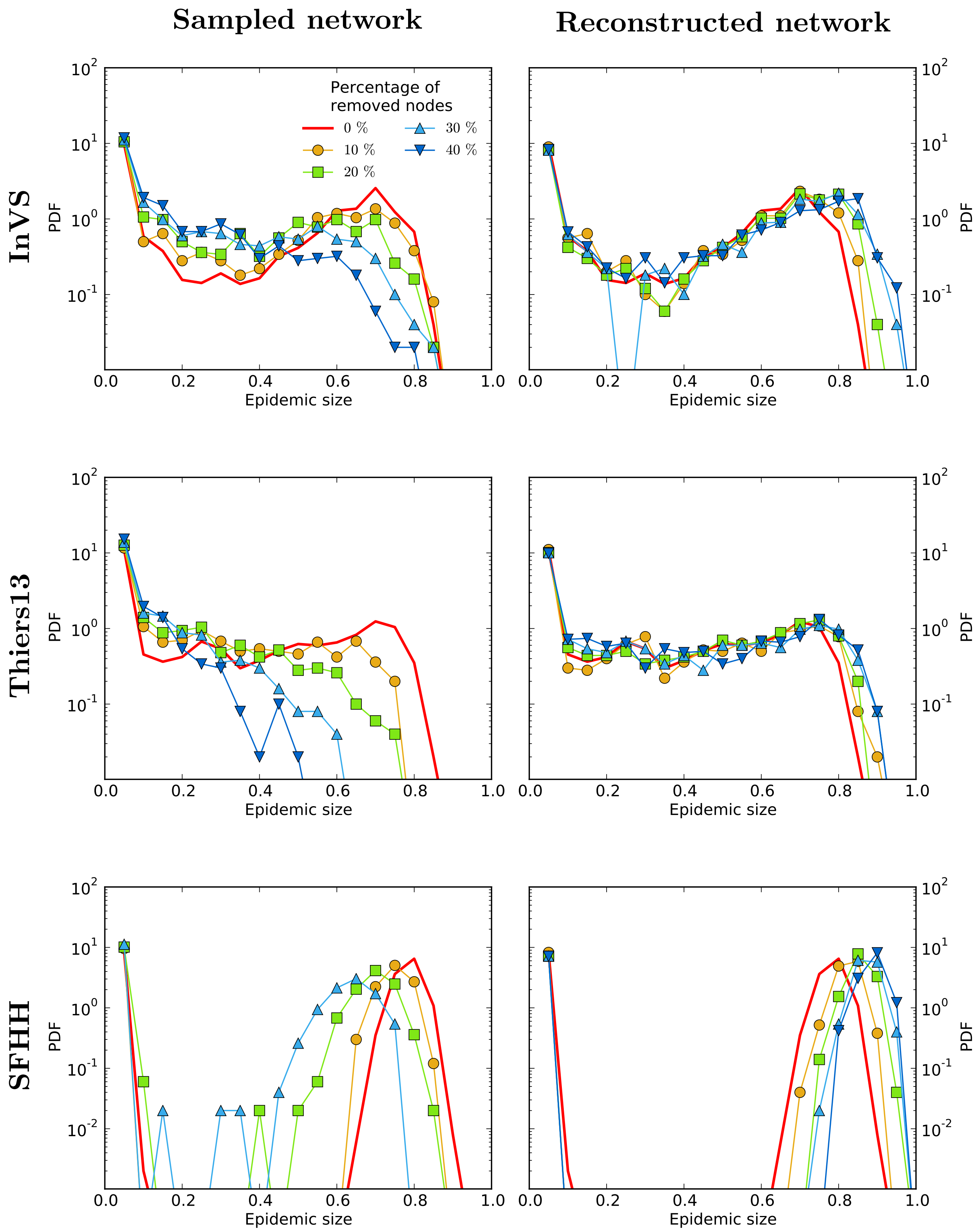

Supplementary Fig. 8. **WST method with constrained transitivity. Comparison of the outcome of SIR epidemic simulations performed on resampled and reconstructed contact networks.** Distribution of epidemic sizes (fraction of recovered individuals) at the end of SIR processes simulated on top of either resampled (left column) or reconstructed (right) contact networks, for different values of the fraction f of nodes removed. The parameters of the SIR models are $\beta = 0.0004$ and $\beta/\mu = 1000$ (*InVS*) or $\beta/\mu = 100$ (*Thiers13* and *SFHH*). The case $f = 0$ corresponds to simulations using the whole data set, i.e., the reference case. For each value of f, $1,000$ independent simulations were performed.

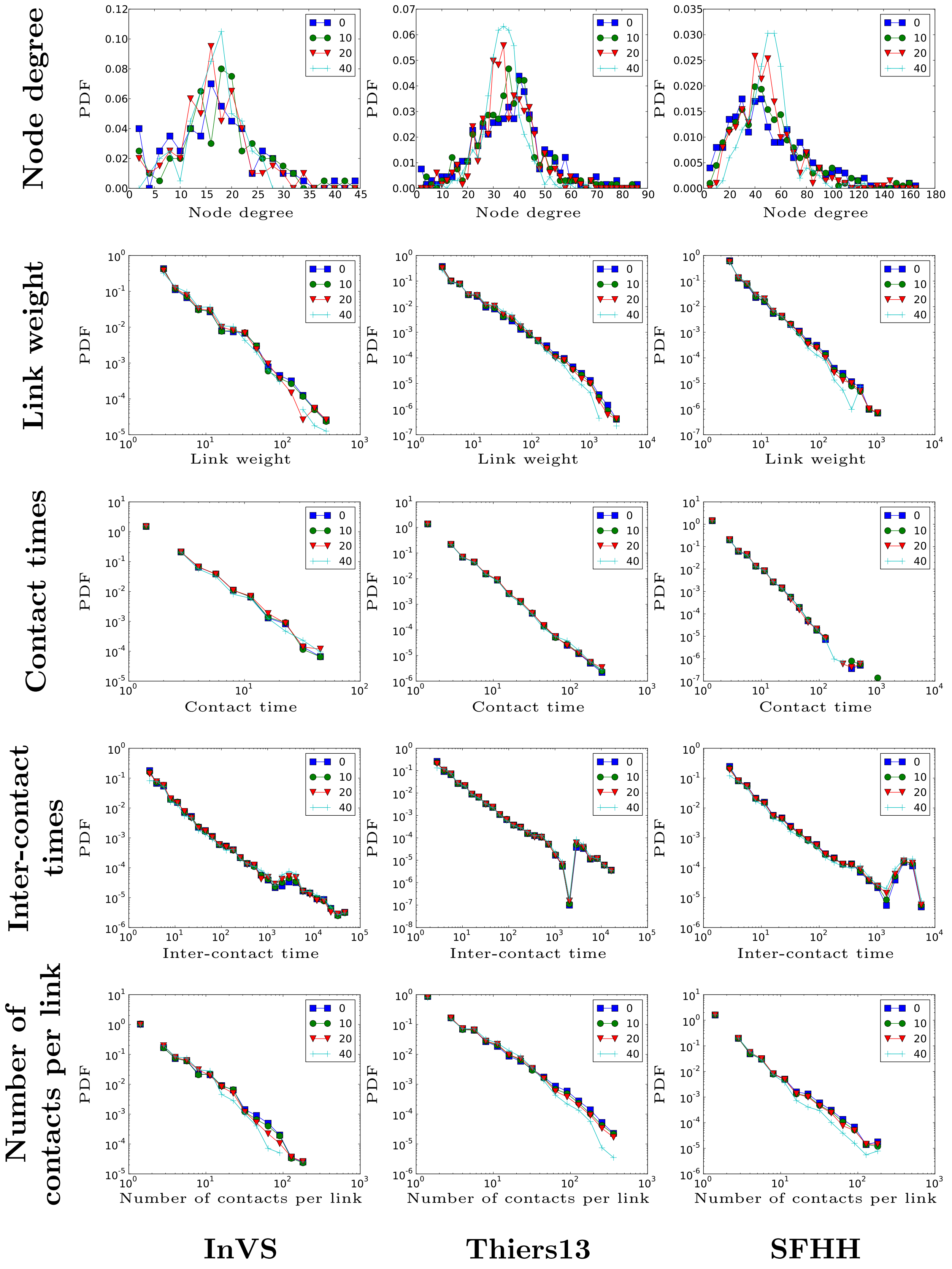

Supplementary Fig. 9. Properties of the reconstructed contact network. Same as Fig. 1 but for the reconstructed networks: Distributions of structural (degrees and weights in the aggregated contact network) and temporal (contact times, inter-contact times, number of contacts per link) properties of the surrogate contact networks, for different fractions f of nodes excluded. For each value of f, the distributions are computed on a single reconstructed network.

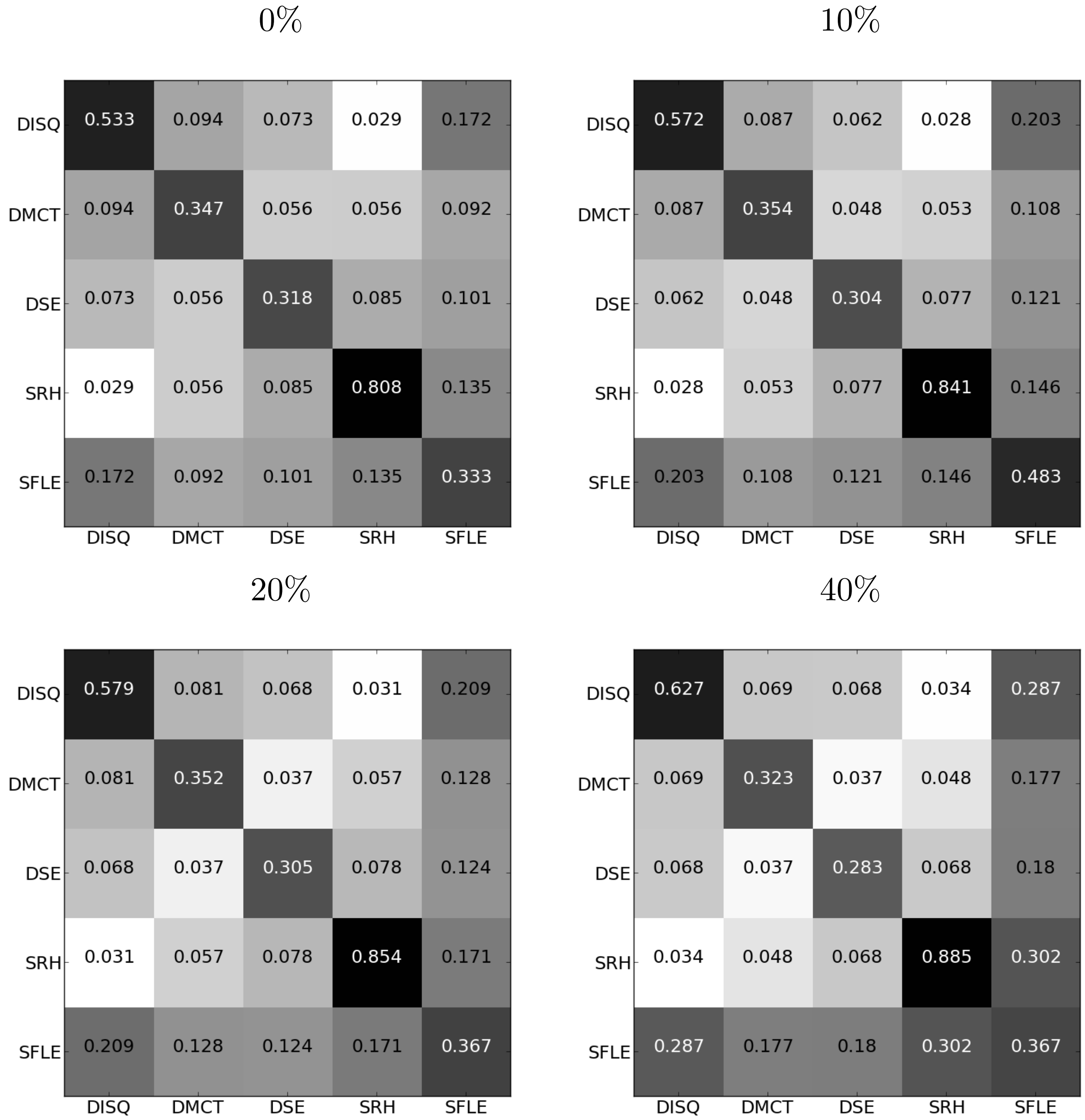

Supplementary Fig. 10. **Properties of the reconstructed contact network: link density contact matrices (*InVS*).** Comparison of link density contact matrices for the reconstructed network of the workplace data, for different values of the fraction f of excluded nodes, with the original one ($f = 0$). For each value of f, each matrix element is an average over 100 realisations of the sampling.

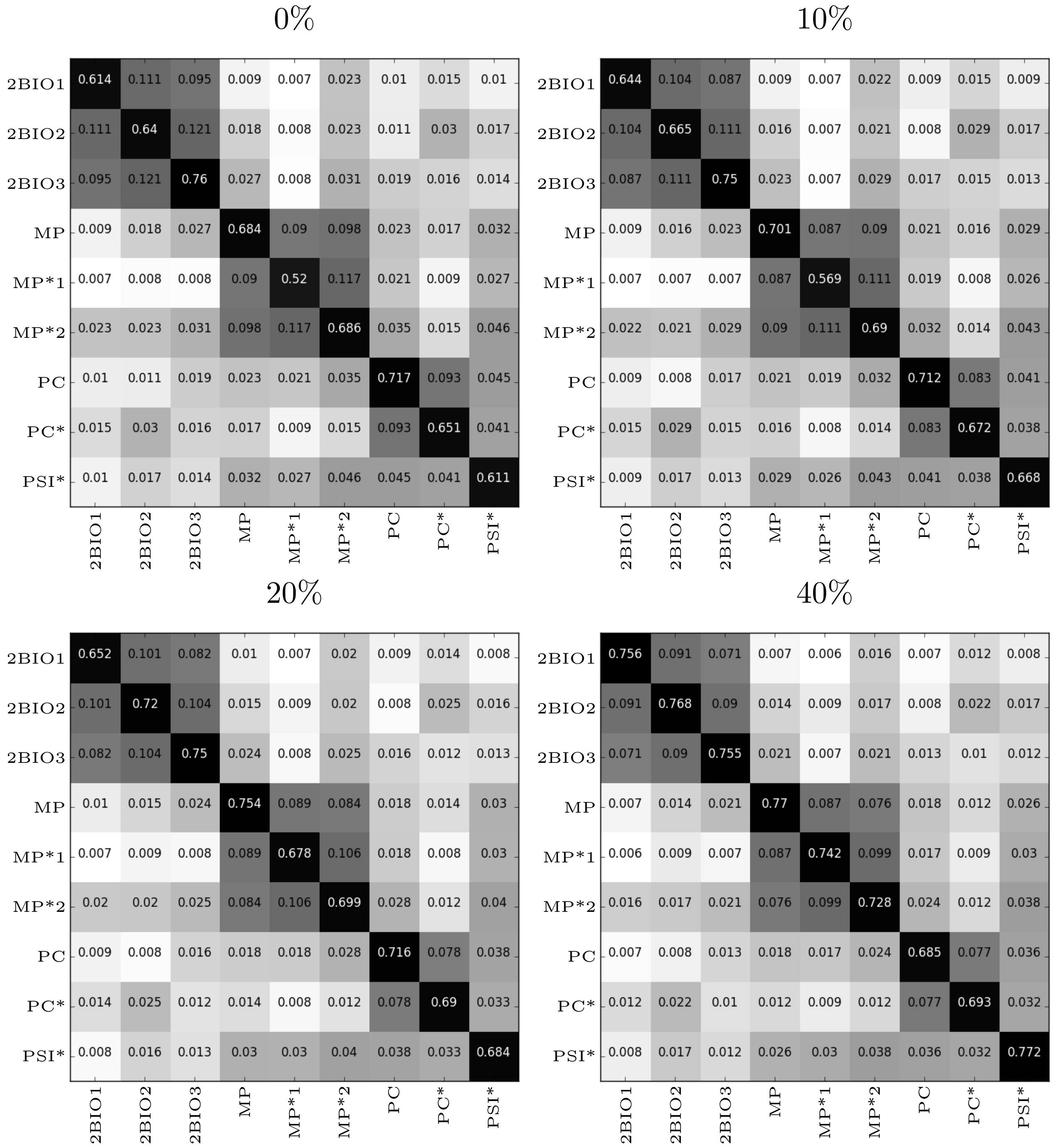

Supplementary Fig. 11. Properties of the reconstructed contact network: link density contact matrices (*Thiers13*). Comparison of link density contact matrices for the reconstructed network of the high school data, for different values of the fraction f of excluded nodes, with the original one ($f = 0$). For each value of f, each matrix element is an average over 100 realisations of the sampling.

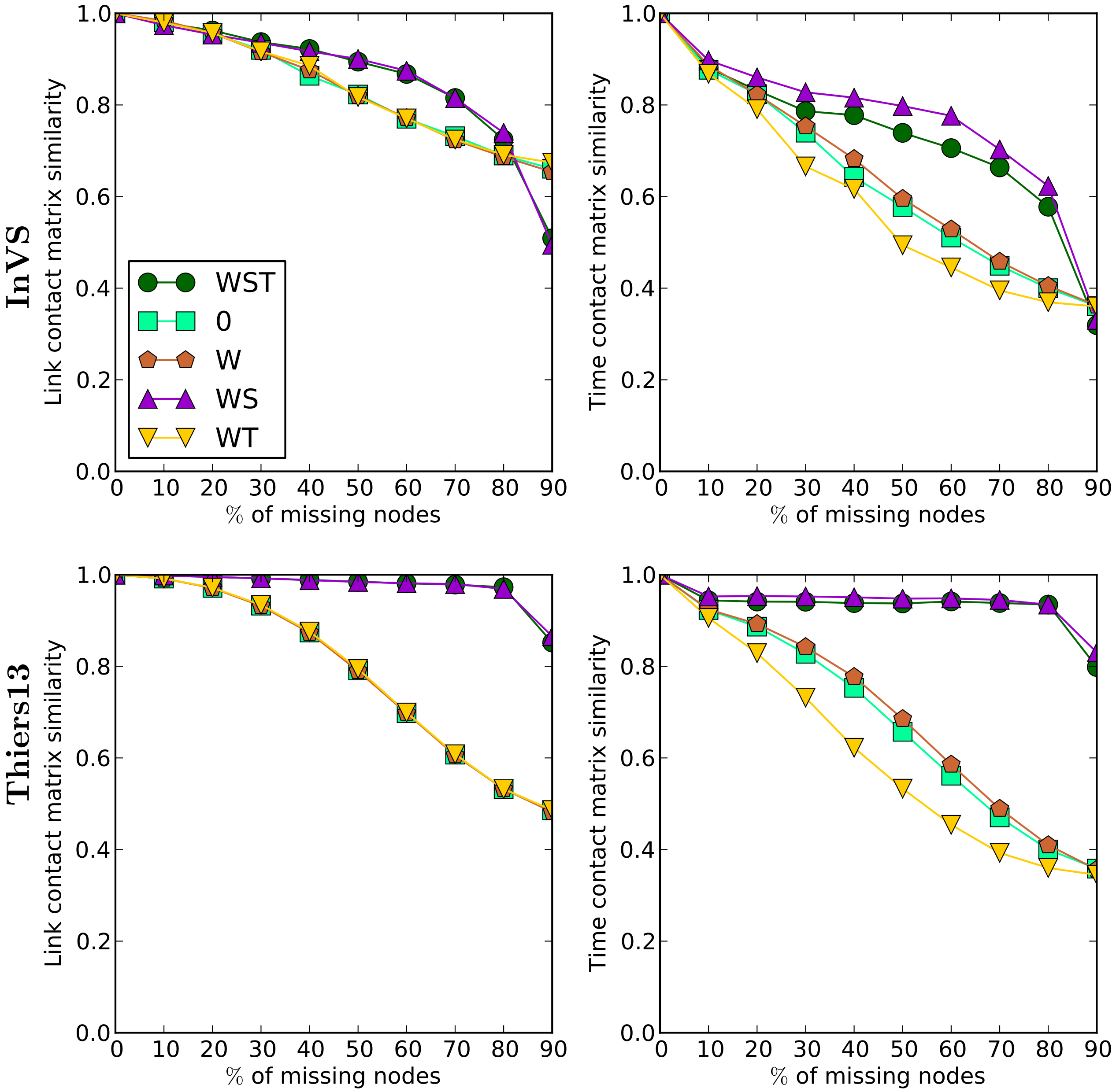

Supplementary Fig. 12. Similarity of contact matrices for different reconstruction methods Median cosine similarity between the link density and contact time density contact matrices computed between the reconstructed network and for the original contact matrices, as a function of the fraction f of removed nodes. For each value of f, the median is computed over 100 realisations of the reconstruction.

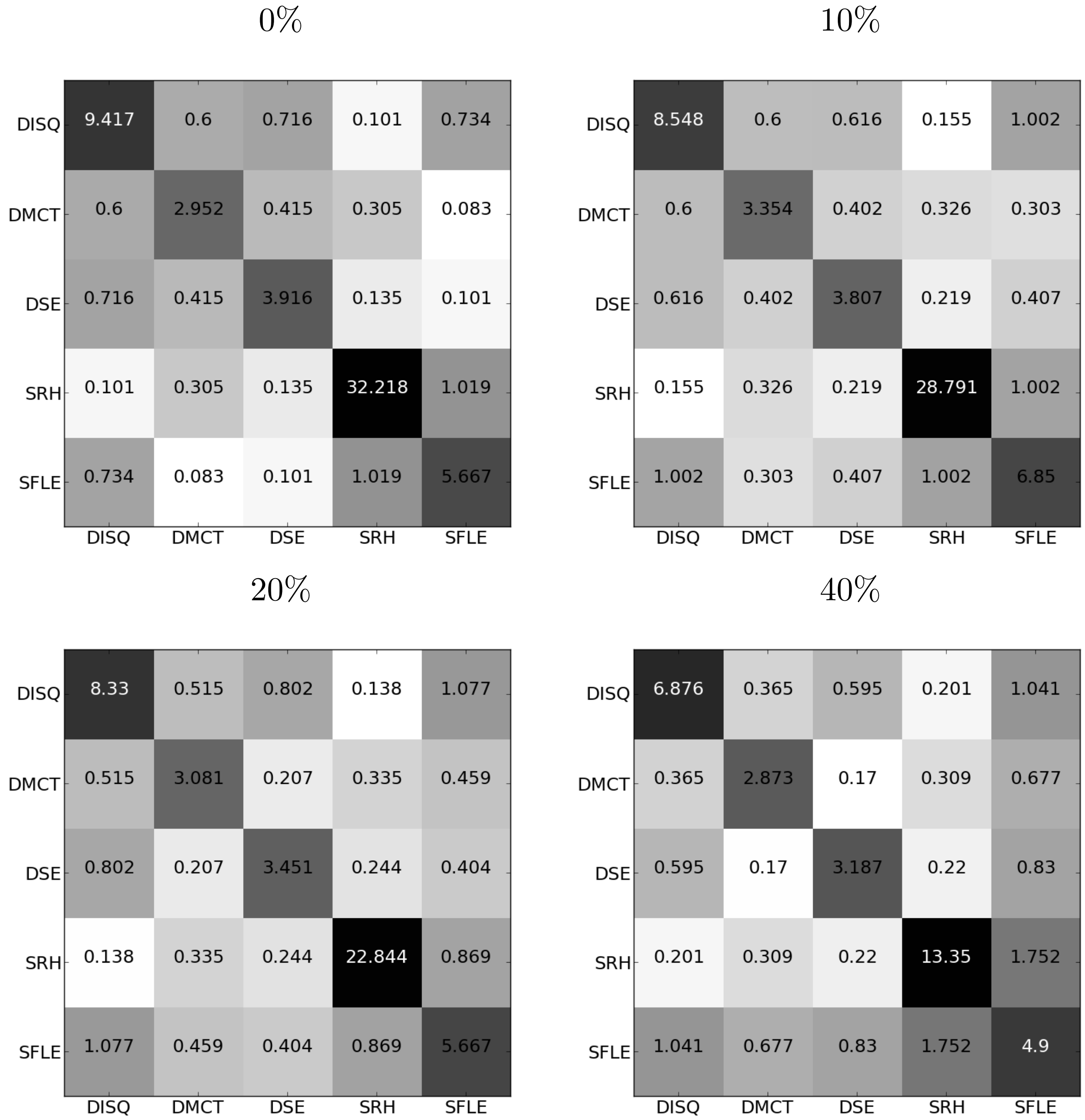

Supplementary Fig. 13. Properties of the reconstructed contact network: time density contact matrices (*InVS*). Comparison of the contact time density contact matrices for the reconstructed network of the workplace data, for different fractions of excluded nodes, f, with the original one ($f = 0$). Each matrix element AB gives the average time spent in contact between a node of department A and a node of department B. For each value of f, each matrix element is an average over 100 realisations of the sampling.

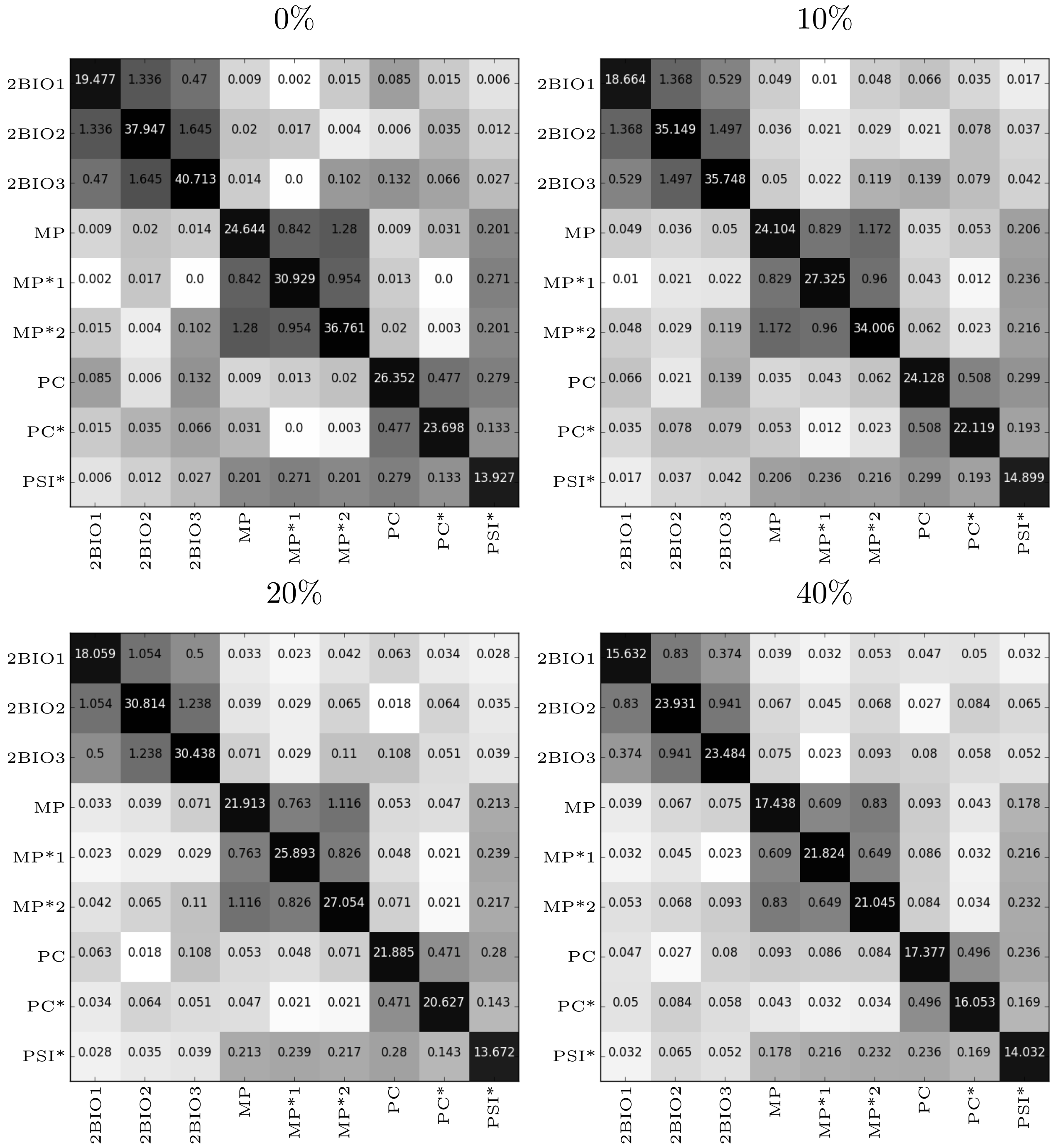

Supplementary Fig. 14. Properties of the reconstructed contact network: time density contact matrices (*Thiers13*). Contact time density contact matrices for the reconstructed network of the high school data, for different fractions of nodes excluded, f. Each matrix element AB gives the average time spent in contact between a node of class A and a node of class B. For each value of f, each matrix element is an average over 100 realisations of the sampling.

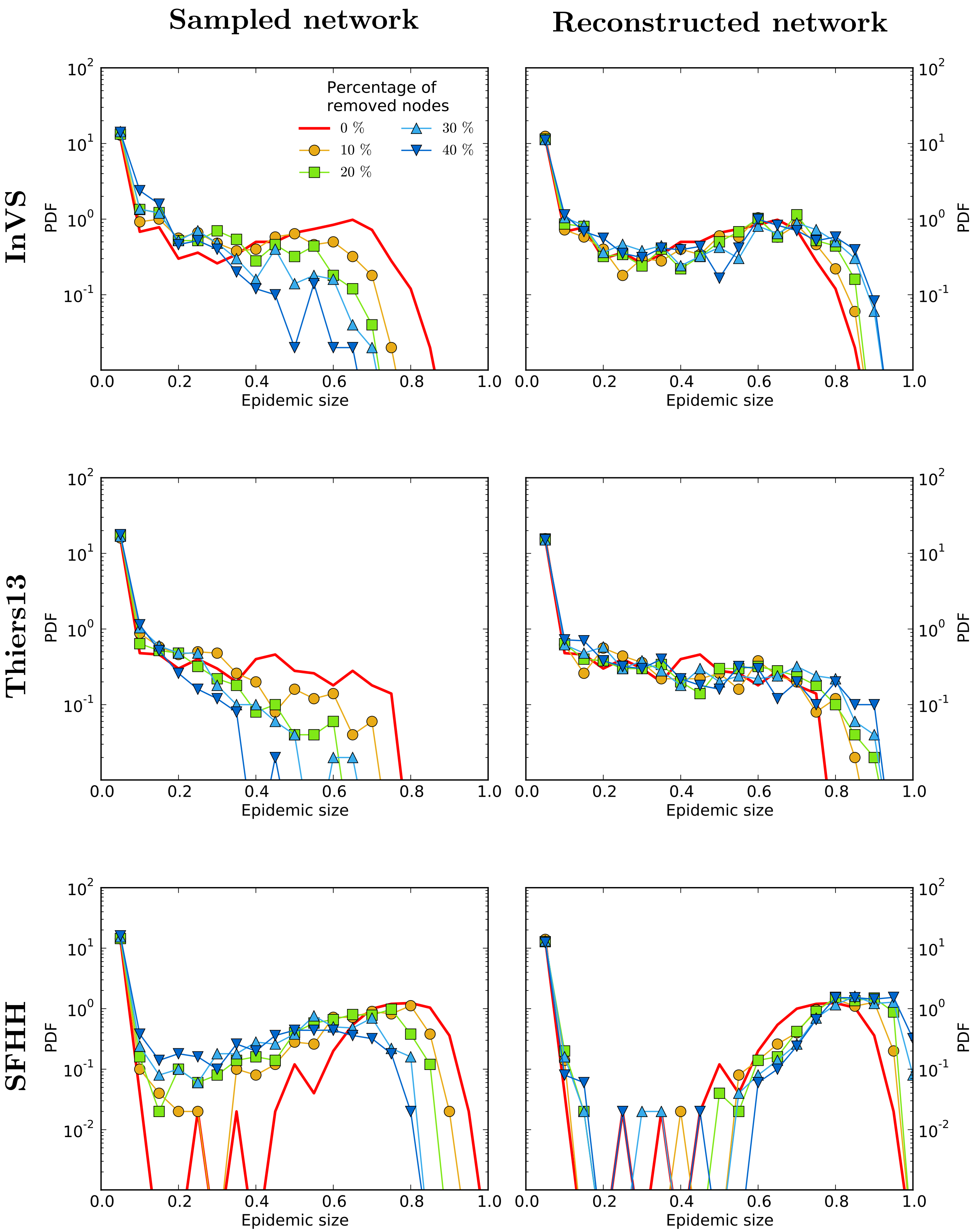

Supplementary Fig. 15. Outcome of SIR epidemic simulations on resampled and reconstructed networks for different parameter values. Distribution of epidemic sizes (fraction of recovered individuals) at the end of SIR processes simulated on top of either resampled (left column) or reconstructed (right) contact networks, using the **WST** method, for different values of the fraction f of nodes removed. The parameters of the SIR models are $\beta = 0.004$ and $\beta/\mu = 1000$ (*InVS*) or $\beta/\mu = 100$ (*Thiers13* and *SFHH*). The case $f = 0$ corresponds to simulations using the whole data set, i.e., the reference case. For each value of f, 1,000 independent simulations were performed.

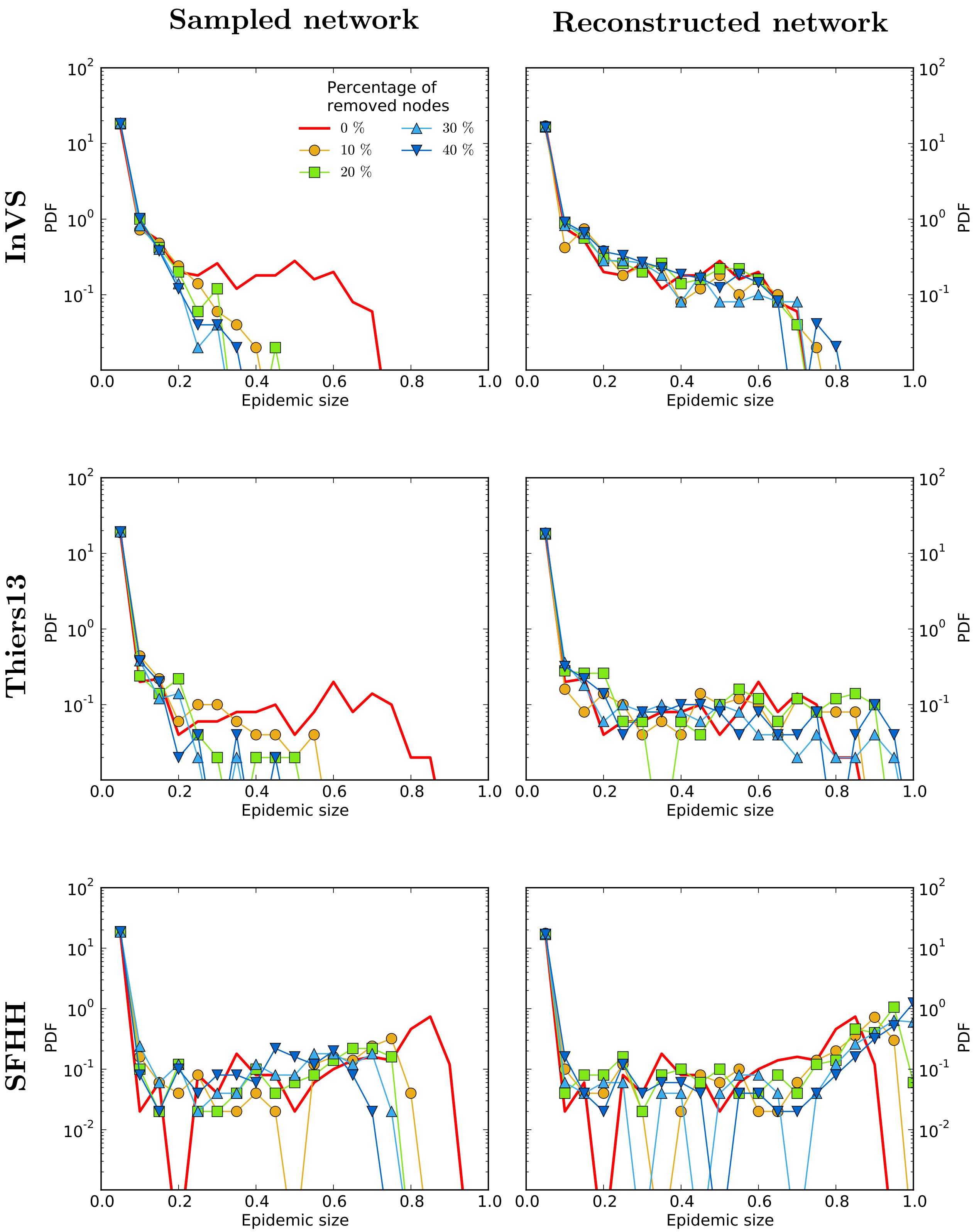

Supplementary Fig. 16. Outcome of SIR epidemic simulations on resampled and reconstructed networks for different parameter values. Distribution of epidemic sizes (fraction of recovered individuals) at the end of SIR processes simulated on top of either resampled (left column) or reconstructed (right) contact networks, using the **WST** method, for different values of the fraction f of nodes removed. The parameters of the SIR models are $\beta = 0.04$ and $\beta/\mu = 1000$ (*InVS*) or $\beta/\mu = 100$ (*Thiers13* and *SFHH*). The case $f = 0$ corresponds to simulations using the whole data set, i.e., the reference case. For each value of f, $1,000$ independent simulations were performed.

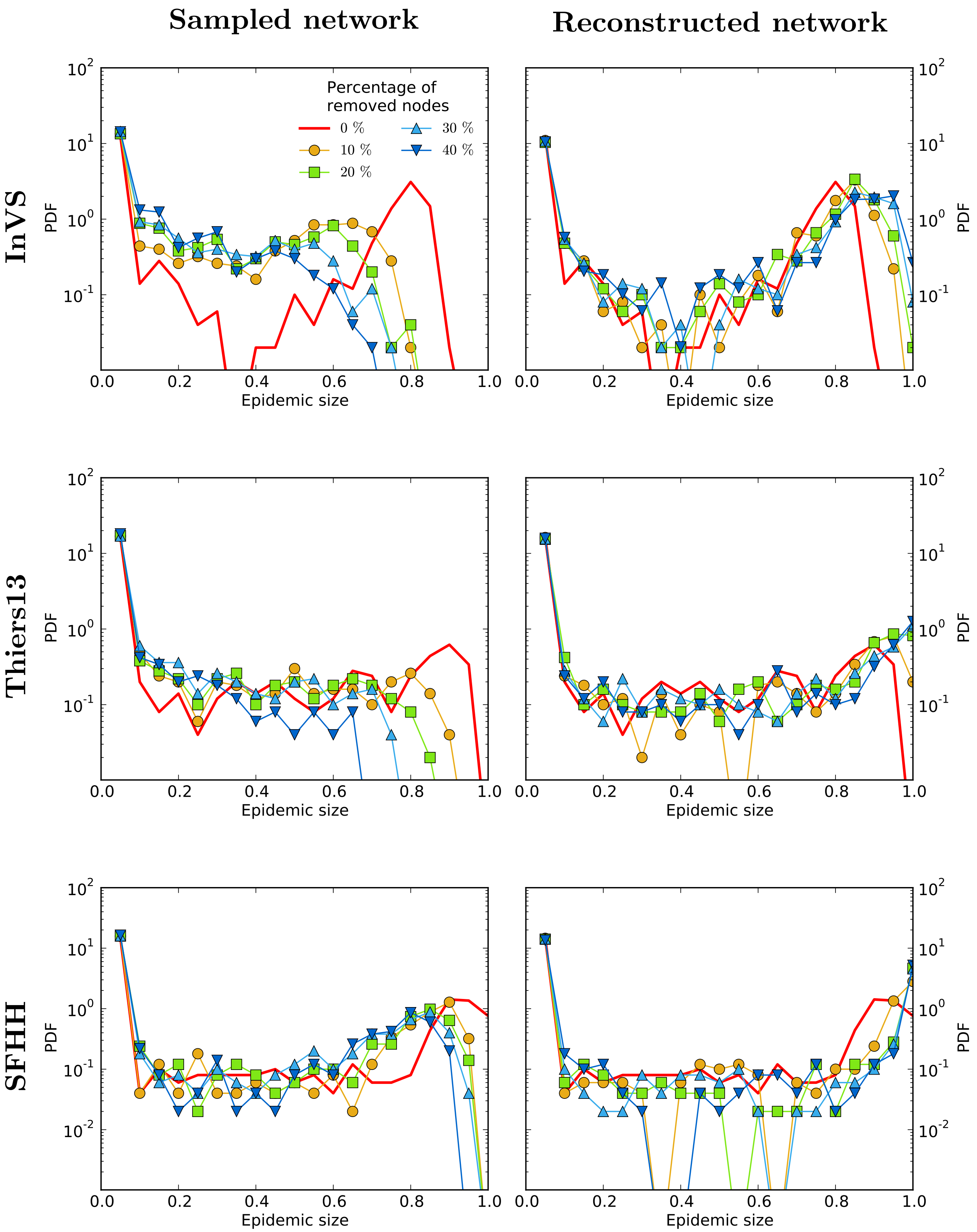

Supplementary Fig. 17. Outcome of SIR epidemic simulations on resampled and reconstructed networks for different parameter values. Distribution of epidemic sizes (fraction of recovered individuals) at the end of SIR processes simulated on top of either resampled (left column) or reconstructed (right) contact networks, using the **WST** method, for different values of the fraction f of nodes removed. The parameters of the SIR models are $\beta = 0.04$ and $\beta/\mu = 4000$ (*InVS*) or $\beta/\mu = 400$ (*Thiers13* and *SFHH*). The case $f = 0$ corresponds to simulations using the whole data set, i.e., the reference case. For each value of f, $1,000$ independent simulations were performed.

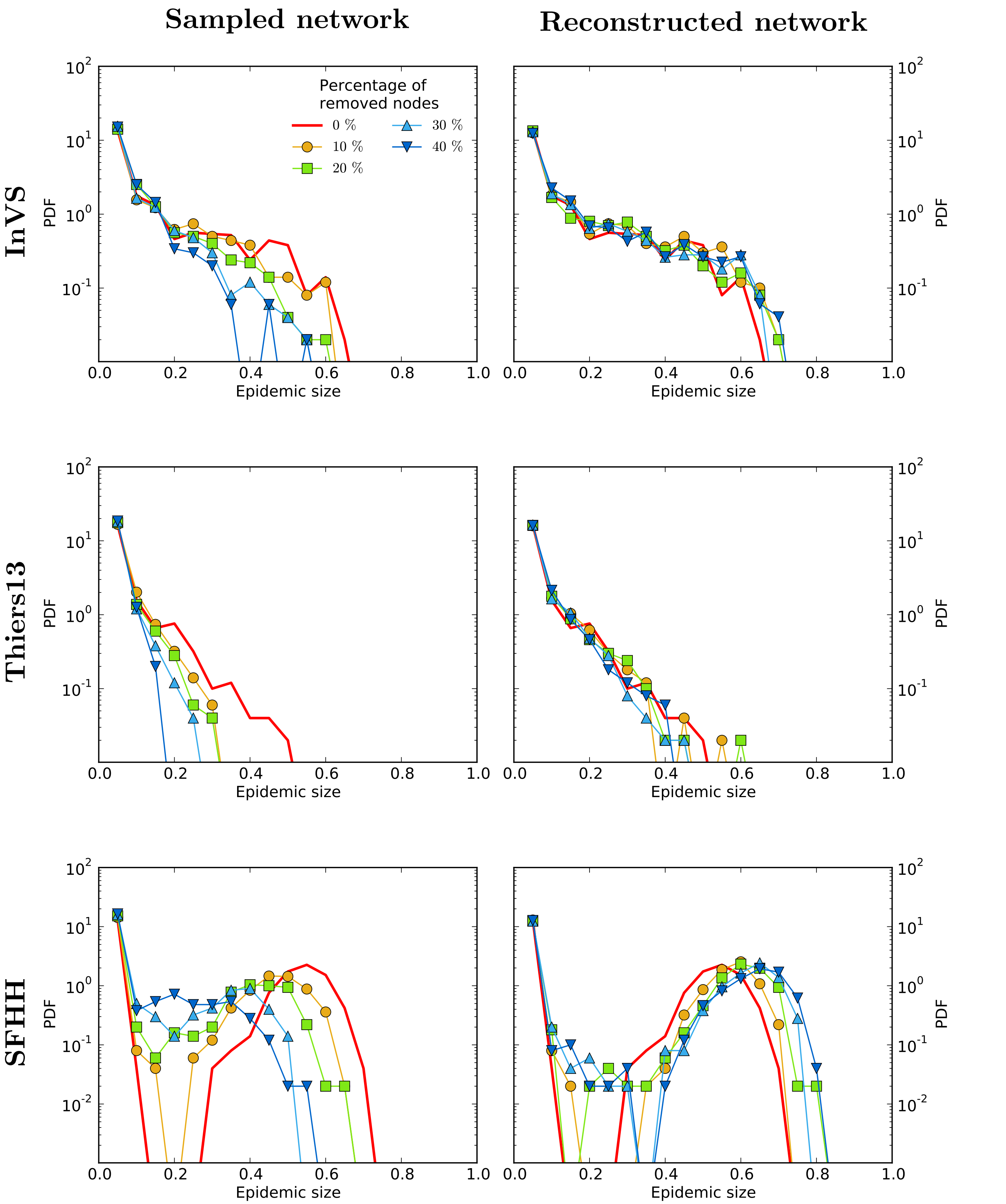

Supplementary Fig. 18. **Outcome of SIR epidemic simulations on resampled and reconstructed networks for different parameter values.** Distribution of epidemic sizes (fraction of recovered individuals) at the end of SIR processes simulated on top of either resampled (left column) or reconstructed (right) contact networks, using the **WST** method, for different values of the fraction f of nodes removed. The parameters of the SIR models are $\beta = 0.0004$ and $\beta/\mu = 500$ (*InVS*) or $\beta/\mu = 50$ (*Thiers13* and *SFHH*). The case $f = 0$ corresponds to simulations using the whole data set, i.e., the reference case. For each value of f, $1,000$ independent simulations were performed.

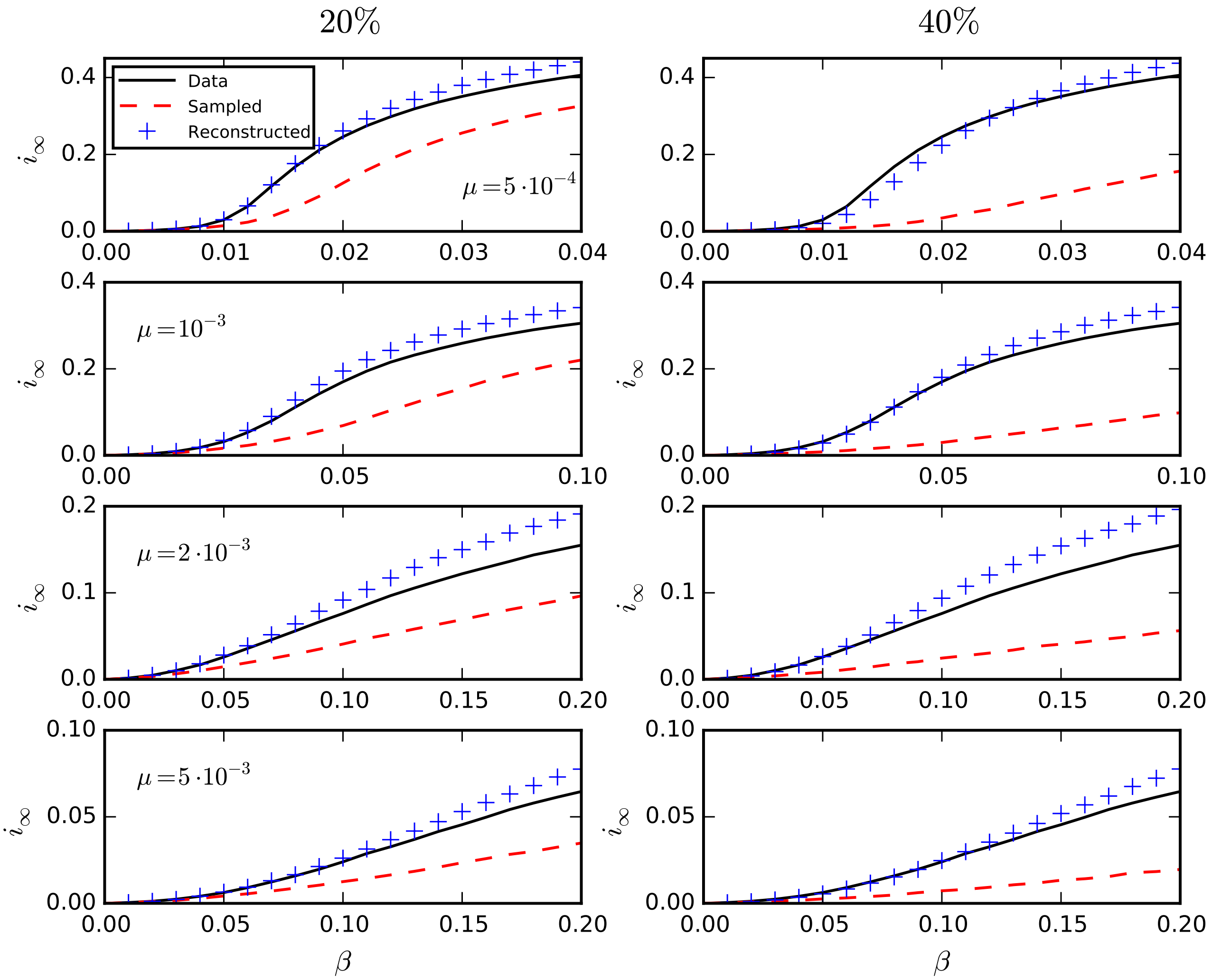

Supplementary Fig. 19. Phase diagram of the SIS model for original, resampled and reconstructed contact networks (*Thiers13* data set). Each panel shows the stationary value i_∞ of the prevalence in the stationary state of the SIS model, computed as described in the Methods section, as a function of β, for several values of μ. Here we consider the example of the *Thiers13* data set. The epidemic threshold corresponds to the transition between $i_\infty = 0$ and $i_\infty > 0$. The prevalence curves are computed in each case using either the whole data set (continuous lines), resampled data (dashed lines) or reconstructed contact networks (pluses). The fraction of excluded nodes in the resampling is $f = 20\%$ for the left column and $f = 40\%$ for the right column.

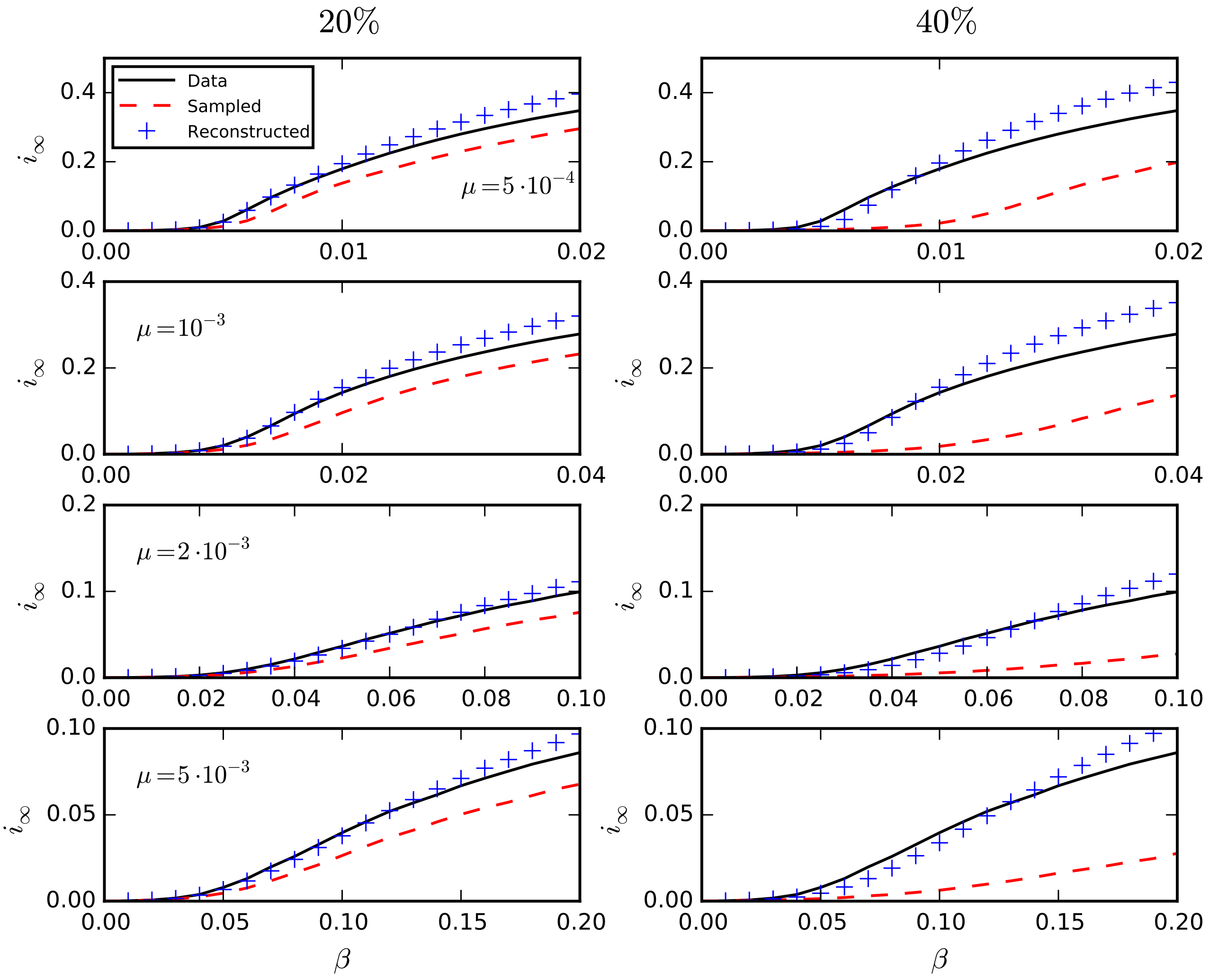

Supplementary Fig. 20. Phase diagram of the SIS model for original, resampled and reconstructed contact networks (*SFHH* data set). Same as Fig. 19 for the *SFHH* (conference) data set.

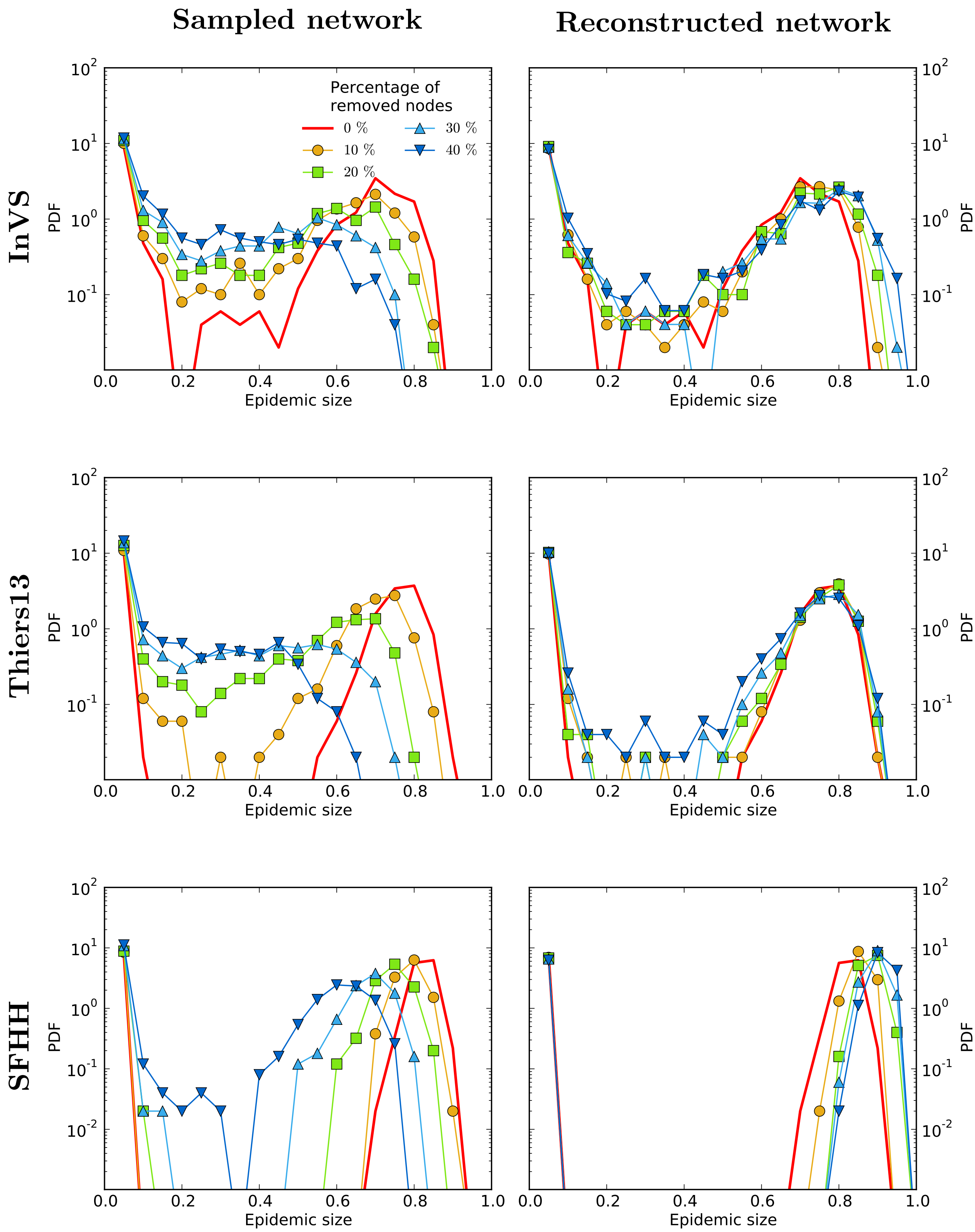

Supplementary Fig. 21. **Method WST on link-shuffled network. Comparison of the outcome of SIR epidemic simulations performed on resampled and reconstructed contact networks.** Distribution of epidemic sizes (fraction of recovered individuals) at the end of SIR processes simulated on top of either resampled (left column) or reconstructed (right) contact networks, for different values of the fraction f of nodes removed. The parameters of the SIR models are $\beta = 0.0004$ and $\beta/\mu = 1000$ (*InVS*) or $\beta/\mu = 100$ (*Thiers13* and *SFHH*). The case $f = 0$ corresponds to simulations using the whole data set, i.e., the reference case. For each value of f, $1,000$ independent simulations were performed.

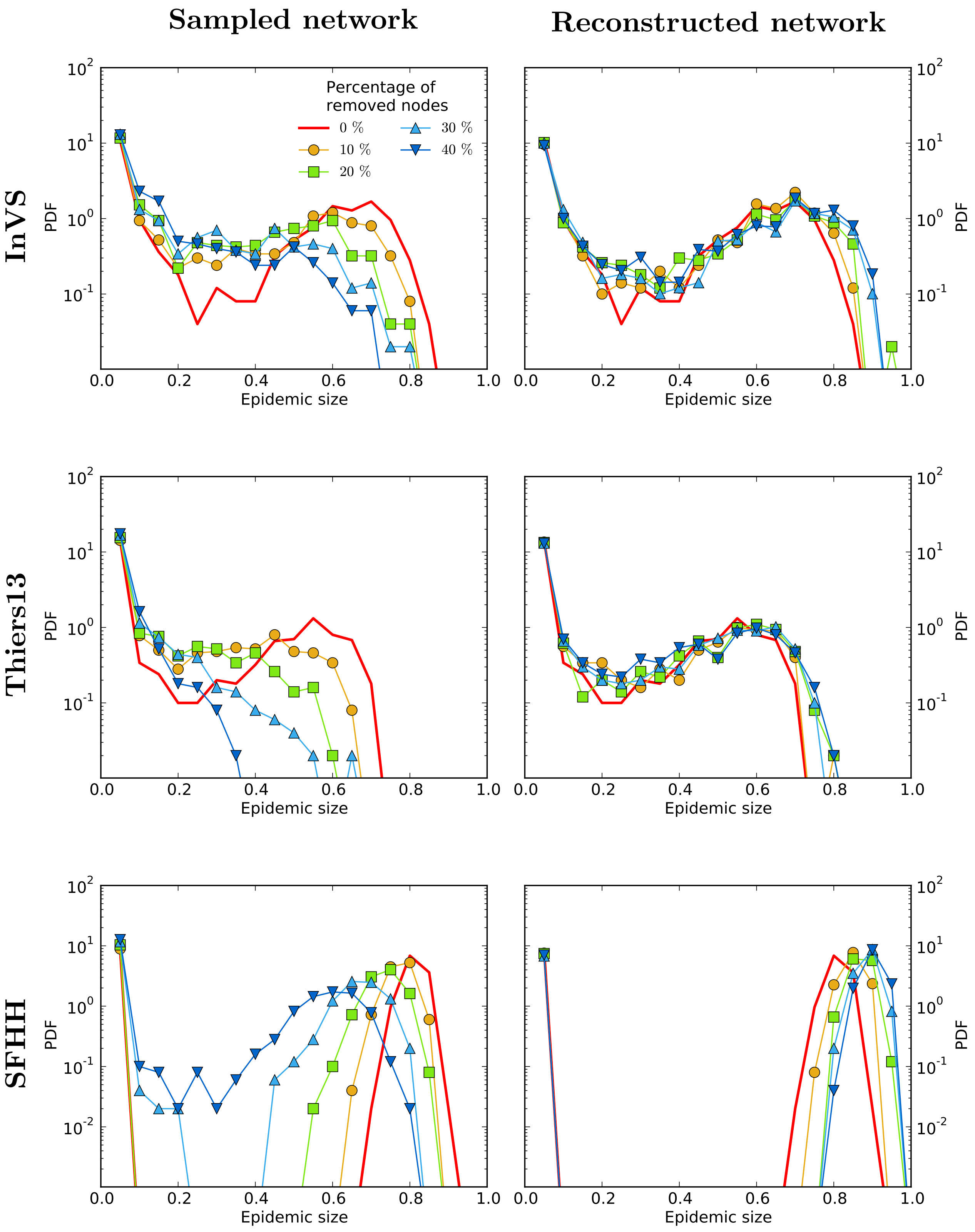

Supplementary Fig. 22. **Method WST on time-shuffled network. Comparison of the outcome of SIR epidemic simulations performed on resampled and reconstructed contact networks.** Distribution of epidemic sizes (fraction of recovered individuals) at the end of SIR processes simulated on top of either resampled (left column) or reconstructed (right) contact networks, for different values of the fraction f of nodes removed. The parameters of the SIR models are $\beta = 0.0004$ and $\beta/\mu = 1000$ (*InVS*) or $\beta/\mu = 100$ (*Thiers13* and *SFHH*). The case $f = 0$ corresponds to simulations using the whole data set, i.e., the reference case. For each value of f, $1,000$ independent simulations were performed.

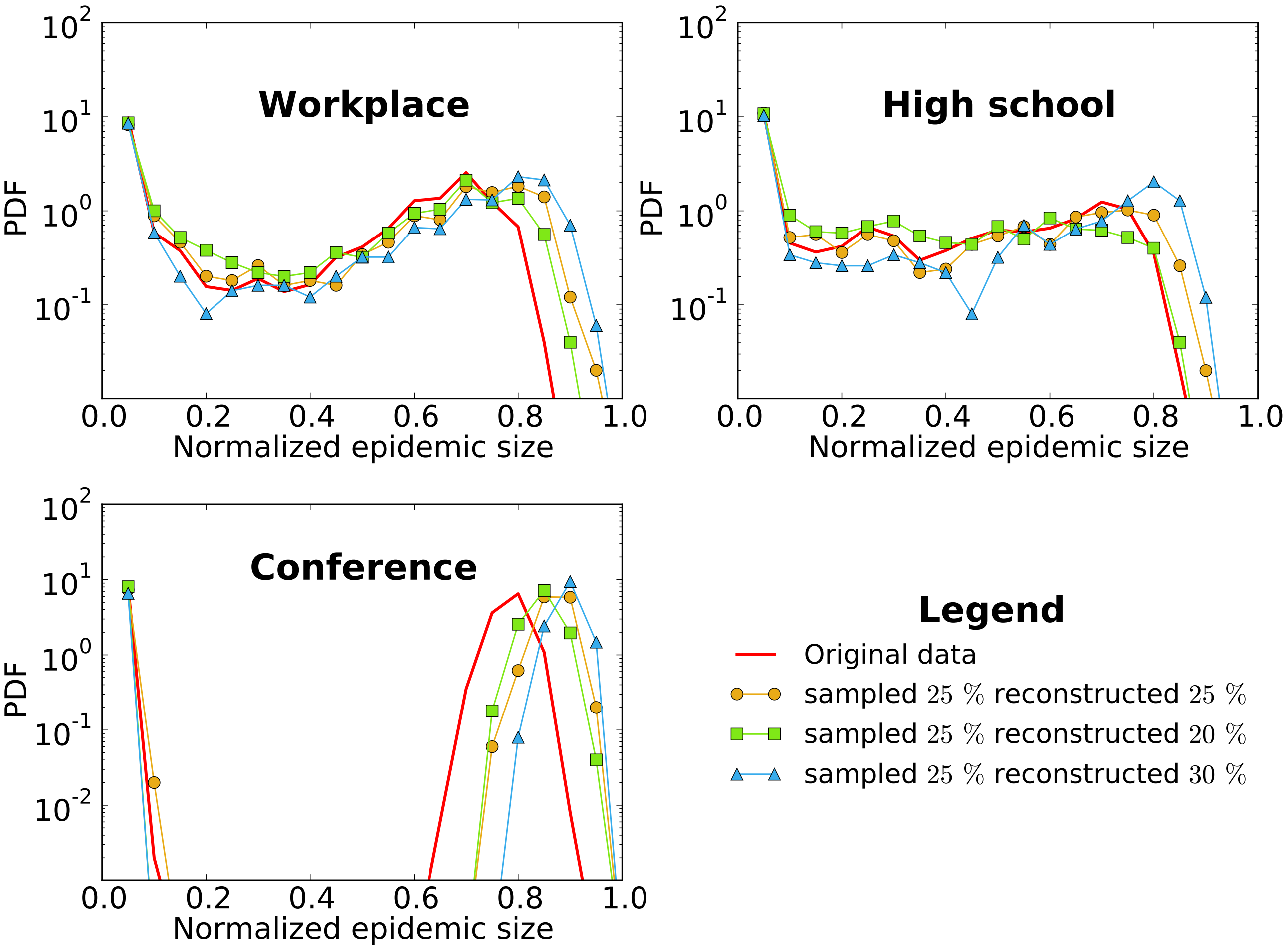

Supplementary Fig. 23. **Uncertainty on the sampling fraction. Comparison of the outcome of SIR epidemic simulations performed on contact networks where 25 % of nodes were removed, and reconstructed with different values of the assumed sampling fraction.** Distribution of epidemic sizes (fraction of recovered individuals) at the end of SIR processes simulated on top of either resampled (left column) or reconstructed (right) contact networks, for different values of the fraction f of nodes removed. The parameters of the SIR models are $\beta = 0.0004$ and $\beta/\mu = 1000$ (*InVS*) or $\beta/\mu = 100$ (*Thiers13* and *SFHH*). The case "Original data" corresponds to simulations using the whole data set, i.e., the reference case. For each value of f, 1,000 independent simulations were performed.

SUPPLEMENTARY NOTES

Supplementary Note 1: Effect of sampling on the temporal network of contacts

As described in the main text, we consider temporally resolved networks of contacts $\mathcal{T}$ in a population $\mathcal{P}$ of N individuals and we perform a resampling experiment by selecting a subpopulation $\tilde{\mathcal{P}}$ of these individuals, of size $\tilde{N} = (1-f)N$. We assume that only the contacts occurring among the subpopulation $\tilde{\mathcal{P}}$ are known and we compare the properties of the corresponding resampled subnetwork $\tilde{\mathcal{T}}$ with those of the original network.

Supplementary Fig. 1 shows how population sampling affects several statistical properties of the contact networks. On the one hand, the degree distribution of the aggregated network of contacts systematically shifts towards smaller degree value. This is expected as each remaining node has in the resampled network a degree which is at most its degree in the original network, and is strictly smaller if some of its neighbours are not part of the resampled population. On the other hand, the statistical distributions of several quantities of interest are not affected by sampling: This is the case of the quantities attached either to single contacts or to single links, namely contact and inter-contact durations, number of contacts per link and link weights (the weight of a link is given by the total duration of the contacts between the two corresponding nodes).

Moreover, as shown in Supplementary Fig. 2, the density of the aggregated network, i.e. the ratio between the number of links and the number of possible links, is on average conserved by the random resampling procedure. It varies however for different realisations of the resampling, and the corresponding variance increases with the fraction f of excluded nodes.

Supplementary Fig. 7 shows how the average clustering coefficient of the aggregated network varies with the resampling: notably, it remains high and close to its original value until large values of f are reached. The transitivity of the network, defined as three times the number of triangles divided by the number of connected triplets (connected subgraphs of 3 nodes and 2 edges), is even less affected than the clustering coefficient by the resampling procedure.

In the case of structured populations, Supplementary Fig. 3 & 4 show that the stability of the resampled network's density holds at the more detailed level of the contact matrices of link densities. In such matrices, the element (i, j) is given by the number of links between individuals of groups i and j, normalised by the total number of possible links between these two groups (if n_i denotes the number of individuals in group i, the number of possible links is equal to $n_i n_j/2$ for $i \neq j$ and to $n_i(n_i-1)/2$ for $i = j$). These figures clearly illustrate how the diagonal and block-diagonal structures are preserved, and Supplementary Fig. 2 gives a quantitative assessment of this stability by showing that the cosine similarity between contact matrices between the resampled and original aggregated contact networks remains high even for when a large fraction of the nodes are excluded.

We moreover illustrate in Supplementary Fig. 5 and 6 the difference in statistical properties of contacts and links within and between groups, still for structured populations:

- the distributions of contact durations are indistinguishable;
- the distribution of link weights (aggregated contact durations) is broader for links between individuals belonging to the same group than for links joining individuals of different groups;
- this is due to the difference in the distributions of numbers of contacts per link, which is broader for links within groups than for links between groups;
- the distributions of inter-contact durations differ also slightly, with smaller averages for within-group links.

Most importantly, all these properties and distributions remain stable under resampling, showing that reliable information on the distributions of contact and inter-contact durations, aggregated contact durations, numbers of contacts per link, can be obtained in the resampled data, including the statistical differences between links joining members of different groups and links between two individuals of the same group.

Supplementary Note 2: Properties of the reconstructed contact networks

As described in the main text and in particular in the Methods section, we construct a surrogate set of contacts concerning the fN individuals excluded by the resampling. We compare here the properties of the resulting contact networks (obtained by merging the resampled contact network $\tilde{\mathcal{T}}$ and the surrogate set of contacts) and of the original contact network, $\mathcal{T}$.

Supplementary Fig. 9 shows that the degree distribution, which is not constrained by the reconstruction procedure, deviates from the original distribution. On the other hand, the distributions of contact durations, inter-contact

durations, number of contacts per link and link weights are preserved. Moreover, the link density contact matrices of the reconstructed networks (Supplementary Fig. 10 and 11) share a high similarity with the original contact matrices, even for high fractions of nodes excluded (Supplementary Fig. 12).

For completeness, we also compute the contact matrices in contact time density (CMT), in which each element (i, j) is given by the total time in contact between individuals of groups i and j, normalised by the total number of possible links between these two groups: it gives the average time spent in contact by two random individuals of groups i and j. Supplementary Fig. 12, 13 and 14 show that the structure of these matrices is well recovered by the reconstruction methods, with high similarity with the original matrices.

SUPPLEMENTARY METHODS

Detailed alternative reconstruction methods

We give here details on the alternative reconstruction methods mentioned in the main text, which use less information than the **WST** method. In each case we consider the same setup as the complete method: a population $\mathcal{P}$ of N individuals (the nodes of the contact network), potentially organised in groups, for which we know all the contacts taking place among a subpopulation $\tilde{\mathcal{P}}$ of size $\tilde{N} = (1-f)N$. For the remaining $n = N - \tilde{N} = fN$ individuals, no contact information is available, but we know to which group they belong. We also have access to the overall activity timeline, *i.e.*, to the successive intervals during which contacts can happen (daytimes), and are excluded (nights and weekends). The alternative reconstruction methods are the following:

0: We perform the reconstruction using only the network density and the average link weight, both measured in the resampled network $\tilde{\mathcal{T}}$. The algorithm goes as follows:

1. we measure in the resampled data:
    - the density ρ of links in the time-aggregated network;
    - the average link weight $\langle w \rangle_\mathrm{s}$ (the weight of a link is defined as the total contact time between the two linked nodes);
2. we compute the number of links e that must be added to keep the network density constant when we add the n excluded nodes;
3. we construct e links according to the following procedure:
    - a node i is randomly chosen from the set $\mathcal{P} \backslash \tilde{\mathcal{P}}$ of excluded nodes;
    - a node j is randomly chosen from the set $\mathcal{P} \backslash \{i\}$ of all other nodes;
    - we compute $n_{ij} = \langle w \rangle_\mathrm{s} / \Delta t$, where $\Delta t = 20s$ is the temporal resolution of the data set, and we randomly choose n_{ij} time windows of length Δt within the activity windows defined by the activity timeline as contact events between i and j.

W: We perform the reconstruction using only the network density and the distribution of link weights, both measured in the resampled network $\tilde{\mathcal{T}}$. The algorithm goes as follows:

1. we measure in the resampled data:
    - the density ρ of links in the time-aggregated network;
    - the list $\{w\}$ of link weights (the weight of a link is defined as the total contact time between the two linked nodes);
2. we compute the number of links e that must be added to keep the network density constant when we add the n excluded nodes;
3. we construct e links according to the following procedure:
    - a node i is randomly chosen from the set $\mathcal{P} \backslash \tilde{\mathcal{P}}$ of excluded nodes;
    - a node j is randomly chosen from the set $\mathcal{P} \backslash \{i\}$ of all other nodes;
    - from $\{w\}$, we draw the weight w_{ij} of the link ij;
    - we compute $n_{ij} = w_{ij} / \Delta t$, where $\Delta t = 20s$ is the temporal resolution of the data set, and we randomly choose n_{ij} time windows of length Δt within the activity windows defined by the activity timeline as contact events between i and j.

WS: We perform the reconstruction using the network density, the distributions of link weights for internal (within groups) and external (between groups) links, and the structure of the aggregated network given by the link density contact matrix, all measured in the resampled network $\tilde{\mathcal{T}}$. The algorithm goes as follows:

1. we measure in the resampled data:
   - the density ρ of links in the time-aggregated network;
   - a *row-normalised* contact matrix C, in which the element C_{AB} gives the probability for a node in group A to have a link to a node of group B;
   - the lists $\{w\}^{\mathrm{int}}$ and $\{w\}^{\mathrm{ext}}$ of link weights for respectively internal and external links (internal links are links between nodes that belong to the same group, external links are links between nodes from different groups);
2. we compute the number of links e that must be added to keep the network density constant when we add the n excluded nodes;
3. we construct e links according to the following procedure:
   - a node i is randomly chosen from the set $\mathcal{P}\backslash\tilde{\mathcal{P}}$ of excluded nodes;
   - knowing the group A that i belongs to, we extract at random a target group B with probability given by C_{AB};
   - we draw a target node j at random from B (if $B = A$, we check that $j \neq i$);
   - depending on whether nodes i and j belong to the same group or not, we draw from $\{w\}^{\mathrm{int}}$ or $\{w\}^{\mathrm{ext}}$ the weight w_{ij} of the link ij;
   - as for the **W** method, we extract at random $w_{ij}/\Delta t$ contact events of length $\Delta t = 20s$ within the activity timeline.

WT: We perform the reconstruction using the network density, the distribution of link weights and the temporal structure of the contacts given by the distributions of contact durations, inter-contact durations, number of contacts per link and initial waiting times before the first contact, all measured in the resampled network $\tilde{\mathcal{T}}$. The algorithm goes as follows:

1. we compute from the activity timeline the time T_{u} as the total duration of the periods during which contacts can occur.
2. we measure in the resampled data:
   - the density ρ of links in the time-aggregated network;
   - the list $\{\tau_{\mathrm{c}}\}$ of contact durations;
   - the list $\{\tau_{\mathrm{ic}}\}$ of inter-contact durations;
   - the list $\{p\}$ of numbers of contacts per link;
   - the list $\{t_0\}$ of initial waiting times before the first contact for each link;
3. we compute the number of links e that must be added to keep the network density constant when we add the n excluded nodes;
4. we construct e links according to the following procedure:
   (a) a node i is randomly chosen from the set $\mathcal{P}\backslash\tilde{\mathcal{P}}$ of excluded nodes;
   (b) a node j is randomly chosen from the set $\mathcal{P}\backslash\{i\}$ of all other nodes;
   (c) we draw from $\{p\}$ the number of contact events p taking place over the link ij;
   (d) from $\{t_0\}$, we draw the initial waiting time t_0 before the first contact;
   (e) from $\{\tau_{\mathrm{c}}\}$, we draw p contact durations τ_{c}^k, $k = 1, \cdots, p$;
   (f) from $\{\tau_{\mathrm{ic}}\}$, we draw $p-1$ inter-contact durations τ_{ic}^m, $m = 1, \cdots, p-1$;
   (g) while $t_0 + \sum_k \tau_{\mathrm{c}}^k + \sum_m \tau_{\mathrm{ic}}^m > T_{\mathrm{u}}$, we repeat steps (c) to (f);
   (h) from t_0,the τ_{c}^k and τ_{ic}^m, we build the contact timeline of the link ij;
   (i) finally, we insert in the contact timeline the breaks defined by the activity timeline.

Reconstruction with fixed transitivity

In order to constrain the transitivity to its value measured in the resampled data, we add to the WST algorithm the following elements:

1. we measure in the resampled data the transitivity σ_0 of the time-aggregated network;
2. for the construction of each link of a node i:
   - we calculate the current transitivity σ of the network;
   - we list the potential targets j in two lists C_Δ and $C_\wedge$, depending on whether the creation of a link between i and j would close a triangle or not;
   -
     – if $\sigma < \sigma_0$, we draw a target node j at random from C_Δ such that i and j are not linked;
     – else if $\sigma > \sigma_0$, we draw a target node j at random from $C_\wedge$ such that i and j are not linked.

We show in Supplementary Fig. 8 the outcome of simulations performed on reconstructed data sets using this modified algorithm.
